# EVA1A (Eva-1 Homolog A) Promotes Endothelial Apoptosis and Inflammatory Activation Under Disturbed Flow Via Regulation of Autophagy

**DOI:** 10.1161/ATVBAHA.122.318110

**Published:** 2023-02-16

**Authors:** Lindsay Canham, Sam Sendac, Mannekomba R. Diagbouga, Elena Wolodimeroff, Daniela Pirri, Blanca Tardajos Ayllon, Shuang Feng, Celine Souilhol, Timothy J.A. Chico, Paul C. Evans, Jovana Serbanovic-Canic

**Affiliations:** 1 Department of Infection, Immunity, and Cardiovascular Disease, INSIGNEO Institute for In Silico Medicine, and the Bateson Centre, University of Sheffield, United Kingdom (L.C., S.S., M.R.D., E.W., B.T.A., S.F., T.J.A.C., P.C.E., J.S.-C.).; 4 William Harvey Research Institute, Barts and the London School of Medicine and Dentistry, Queen Mary University of London, United Kingdom (P.C.E.).; 2 National Heart and Lung Institute, Imperial College London, United Kingdom (D.P.).; 3 Biomolecular Sciences Research Centre, Sheffield Hallam University, United Kingdom (C.S.).

**Keywords:** apoptosis, atherosclerosis, autophagy, endothelial cell, zebrafish

## Abstract

Hemodynamic wall shear stress (WSS) exerted on the endothelium by flowing blood determines the spatial distribution of atherosclerotic lesions. Disturbed flow (DF) with a low WSS magnitude and reversing direction promotes atherosclerosis by regulating endothelial cell (EC) viability and function, whereas un-DF which is unidirectional and of high WSS magnitude is atheroprotective. Here, we study the role of EVA1A (eva-1 homolog A), a lysosome and endoplasmic reticulum-associated protein linked to autophagy and apoptosis, in WSS-regulated EC dysfunction.

**Methods:**

The effect of WSS on EVA1A expression was studied using porcine and mouse aortas and cultured human ECs exposed to flow. EVA1A was silenced in vitro in human ECs and in vivo in zebrafish using siRNA (small interfering RNA) and morpholinos, respectively.

**Results:**

EVA1A was induced by proatherogenic DF at both mRNA and protein levels. *EVA1A* silencing resulted in decreased EC apoptosis, permeability, and expression of inflammatory markers under DF. Assessment of autophagic flux using the autolysosome inhibitor, bafilomycin coupled to the autophagy markers LC3-II (microtubule-associated protein 1 light chain 3-II) and p62, revealed that *EVA1A* knockdown promotes autophagy when ECs are exposed to DF, but not un-DF . Blocking autophagic flux led to increased EC apoptosis in *EVA1A*-knockdown cells exposed to DF, suggesting that autophagy mediates the effects of DF on EC dysfunction. Mechanistically, *EVA1A* expression was regulated by flow direction via TWIST1 (twist basic helix-loop-helix transcription factor 1). In vivo, knockdown of *EVA1A* orthologue in zebrafish resulted in reduced EC apoptosis, confirming the proapoptotic role of EVA1A in the endothelium.

**Conclusions:**

We identified EVA1A as a novel flow-sensitive gene that mediates the effects of proatherogenic DF on EC dysfunction by regulating autophagy.

**Highlights**

- Autophagy-associated protein EVA1A (eva-1 homolog A) is induced by disturbed, proatherogenic flow in mouse, porcine, and human endothelial cells.
- EVA1A accumulation under disturbed flow blocks autophagic flux and promotes endothelial apoptosis and inflammatory activation.
- Changes in flow direction induce EVA1A expression in endothelial cells.
- EVA1A is a downstream target of transcription factor TWIST1 (twist basic helix-loop-helix transcription factor 1).

Atherosclerosis is a chronic inflammatory arterial disease and the main cause of myocardial infarction and stroke. Despite systemic risk factors, atherosclerotic lesions are localized to specific regions of arteries such as branches, bends, and bifurcations.^1,2^ The focal nature of atherosclerosis is mainly determined by the hemodynamic forces exerted on endothelial cells (ECs) lining blood vessels, including wall shear stress (WSS), the frictional force per unit area whose direction is parallel to blood flow. ECs in the athero-prone regions of arteries are exposed to disturbed flow (DF) characterized by WSS of low magnitude and temporal and spatial changes in flow direction. These conditions promote EC inflammation and apoptosis, leading to increased vascular permeability and expression of cell adhesion molecules, such as SELE (E-selectin), ICAM1 (intercellular adhesion molecule 1), and VCAM1 (vascular cell adhesion molecule 1).^3,4^ The resulting accumulation of cells and lipids in the intima lead to formation of atherosclerotic plaques. Conversely, un-DF (UF) characterized by unidirectional WSS of physiologically high magnitude promotes EC quiescence and is atheroprotective.^5^ Mechanisms involved in differential responses of ECs to different types of WSS have been extensively studied but are still not completely understood.^1,5–7^

EVA1A (eva-1 homolog A, also known as FAM176A [family with sequence similarity 176, member A] and TMEM166 [transmembrane protein 166]) was first identified as a lysosome and endoplasmic reticulum-associated protein which colocalizes with autophagosome and promotes apoptosis and autophagy.^8^ Its expression is downregulated in several cancers^9^ and overexpression of EVA1A can inhibit cancer cell growth by inducing autophagy and apoptosis.^10–12^ Conversely, there are examples where EVA1A is ectopically expressed in cancer cells, such as pancreatic^13^ and thyroid cancer.^14^ Additionally, EVA1A promotes resistance to chemotherapeutic agent oxaliplatin in hepatocellular carcinoma via a microRNA miR-125b dependent mechanism.^15^ In the nervous system, EVA1A regulates embryonic neurogenesis and neuronal differentiation by modulating autophagy.^16,17^ Recent studies have demonstrated a role for EVA1A in the cardiovascular system. Cardiomyocyte-specific knockout of EVA1A in mice led to rapid heart failure by impairing autophagy.^18^ EVA1A may also be involved in regulation of plaque stability as increased *EVA1A* mRNA expression was found in symptomatic compared to asymptomatic human carotid plaques.^19^ Whole-body EVA1A knockout in mice was protective against atherosclerosis^20^; however, the underlying mechanism including the potential role of autophagy regulation is incompletely understood. Here, we sought to investigate the role of EVA1A in WSS-regulated endothelial dysfunction and regulation of autophagy in ECs exposed to flow.

## Methods

### Data Availability

The majority of data supporting the findings of this study are presented within the article and its Supplemental Material. Data that are not directly included are available from the corresponding author upon reasonable request.

### Isolation of ECs From Pig Aortas

Pig aortas from 4 to 6 months old animals were obtained immediately after slaughter from a local abattoir and no ethical approval was required. They were cut longitudinally along the outer curvature to expose the lumen. ECs exposed to UF (=outer curvature and descending aorta) or DF (=inner curvature) were harvested by gentle scraping following incubation with collagenase (Sigma; 1 mg/mL in DMEM for 10 minutes at room temperature). Total RNA was isolated using RNeasy Mini Kit (74104, Qiagen), following manufacturer’s instructions.

### Mice

All animal care and experimental procedures conformed to the institution’s ethical requirements, UK Home Office regulations, and guidelines from Directive 2010/63/EU of the European Parliament on the protection of animals used for scientific purposes. Animal care and experimental procedures were carried out under Project Licence P5395C858 issued by the UK Home Office. C57BL/6J mice were housed under specific pathogen-free conditions. Mice aged 8 weeks were used for tissues.

### En Face Staining of Murine Endothelium

The expression levels of EVA1A were assessed in ECs at regions of the inner curvature (DF site), outer curvature (UF site) of murine aortic arch, or the descending aorta (UF site) by en face staining. All antibodies used are listed in the Major Resources Table. Animals were sacrificed by a single intraperitoneal injection of sodium pentobarbital (800 mg/kg) and aortas were perfused in situ with PBS and then perfusion-fixed with 4% Paraformaldehyde before harvesting. Fixed aortas were tested by immunostaining using a rabbit polyclonal EVA1A antibody, whereas ECs were identified by costaining using an anti-CD31 antibody. Nuclei were identified using To-Pro-3 (ThermoFisher). Stained vessels were mounted in ProLong Gold (ThermoFisher) before visualization of endothelial surfaces en face using confocal microscopy (Olympus FV1000 confocal microscope). The expression of EVA1A at each aortic site was assessed by quantification of the mean fluorescence intensities using Fiji software.

### EC Culture and Exposure to WSS

Human umbilical vein ECs (HUVECs) and human aortic ECs (HAECs) from single donors were purchased from PromoCell and cultured according to the manufacturer’s recommendations. Experiments were performed using cells from minimum of 3 different individual donors that were not pooled. ECs at passage 3 to 5 were cultured until confluent in 6 well plates and exposed to flow for 72 hours using an orbital shaking platform (PSU-10i; Grant Instruments) housed inside a cell culture incubator. The radius of orbit of the orbital shaker was 10 mm and the rotation rate was set to 210 revolutions per minute. These conditions generate DF conditions in the center of the well, with low magnitude of WSS (≈5 dynes/cm^2^) and rapid variations in direction, and UF conditions in the periphery of the well with high magnitude of WSS (≈10 dynes/cm^2^) and relatively uniform direction.^21^ To study VCAM1 protein expression, ECs were stimulated with TNF (tumor necrosis factor; PHC3015; Gibco) at concentration of 10 ng/mL for the last 4 hours of flow, as described previously.^22^ To study autophagic flux in ECs exposed to flow, the cells were treated with 50 nM bafilomycin A1 (Sigma) or 0.05% DMSO as control. Bafilomycin was added to the cells for the last 4 hours of 72 hours of flow exposure. For RNA and protein expression analysis, cells were harvested by scraping from specific regions of the well using a standardized template to identify the DF and UF regions. The central DF region was circular and had a radius of 5 mm, whereas the UF region in the periphery of the well had a ring shape with a width of 8 mm, which is based on the computational fluid dynamics model.^21^ HAECs at passage 3 to 5 were seeded onto gelatin-coated Ibidi µ-Slides I^0.4^ (Luer ibiTreat; ibidi) and used when fully confluent. Flowing medium was then applied using the Ibidi pump system to generate high laminar (13 dynes/cm^2^), low laminar (4 dynes/cm^2^), or low oscillatory WSS. For low oscillatory WSS, HAECs were exposed to a repeated cycle of 2 hours of oscillatory flow (±4 dynes/cm^2^, 1 Hz), followed by 10 minutes of unidirectional flow (+4 dynes/cm^2^), to ensure redistribution of nutrients.^23^ The slides and pump apparatus were placed in a cell culture incubator at 37 °C.

### Gene Silencing

HUVEC and HAEC cultures were transfected with siRNA (small interfering RNA) sequences that are known to silence *EVA1A* (L-014799-02; Dharmacon), *TWIST1* (twist basic helix-loop-helix transcription factor 1; L-006434-00; Dharmacon), or with nontargeting control siRNA pool (D-001810-10; Dharmacon), following manufacturer’s instructions. The cells were transfected using Neon Transfection System (Invitrogen; HUVEC) or Lipofectamine RNAiMAX transfection reagent (ThermoFisher; HAEC) and following the manufacturer’s protocols. Final concentrations of 50 nM siRNA (HUVEC) or 20 nM siRNA (HAEC) were used for the experiments. Following transfection, the cells were incubated in complete growth medium for 24 hours (HUVEC) or 6 hours (HAEC) before exposure to flow.

### Immunofluorescent Staining of Cultured ECs

ECs were fixed with paraformaldehyde (4%) and permeabilized with Triton X-100 (0.1%) in PBS. Following blocking with 5% goat serum in PBS for 1 hour, monolayers were incubated for 16 hours at 4 °C with primary antibodies against EVA1A, active caspase 3 (apoptosis marker), PCNA (proliferating cell nuclear antigen; proliferation marker), and CDH5 (cadherin 5; endothelial marker). All antibodies used are listed in the Major Resources Table. This was followed by incubation with appropriate AlexaFluor488- or Alexafluor568-conjugated secondary antibodies (ThermoFisher) for 2 hours at room temperature. Nuclei were identified using DAPI (4′,6-diamidino-2-phenylindole; Sigma). Images were taken with a widefield fluorescence microscope (Leica DMI4000B) and analyzed using Fiji software to calculate the frequency of positive cells. Isotype controls and omission of the primary antibody were used to control for nonspecific staining.

### Quantitative Real-Time Polymerase Chain Reaction

RNA was extracted using the RNeasy Mini Kit (74104; Qiagen) and reverse transcribed into cDNA using the iScript cDNA synthesis kit (1708891; Bio-Rad). Quantitative real-time polymerase chain reaction (qRT-PCR) was used to assess the levels of transcripts with gene-specific primers (Table S1). Reactions were prepared using SsoAdvanced universal SYBR Green supermix (172-5271; Bio-Rad) and following the manufacturer’s instructions and were performed in triplicate. Expression values were normalized against the housekeeping gene (pig *B2M*, human *HPRT*, or zebrafish *bact2*). Fold differences were calculated using the ΔΔCt (delta-delta cycle threshold) method.

### Assay of Permeability

The permeability of EC monolayers exposed to flow was determined using rhodamine-labeled albumin and Transwell inserts, as previously described.^24^ Computational fluid dynamics model showed that the cells are exposed to time-averaged WSS of ≈2 dynes/cm^2^, which is relatively constant across the Transwell.^24^ HUVECs were cultured in 6-well Transwell inserts (CC401; Corning) and then exposed to orbital shaking at 150 revolutions per minute or static conditions for 72 hours. The media in the upper compartment was then replaced with 10% serum-supplemented DMEM containing 1% BPA (bovine plasma albumin) and rhodamine-labeled albumin (1 mg/mL). Media in the lower compartment was sampled at 1 hour and fluorescence was measured using a fluorimeter (Varioskan; Thermoscientific) with excitation at 570 nm and emission at 600 nm. Rhodamine-albumin concentration was calculated using a standard curve.

### Western Blotting

Total cell lysates were isolated using lysis buffer (containing 2% SDS, 10% glycerol, and 5% β-mercaptoethanol). Western blotting was carried out using specific antibodies against calnexin (housekeeper), VCAM1, LC3 (microtubule-associated protein 1 light chain 3-II), and p62. All antibodies used are listed in the Major Resources Table. Horseradish peroxidase-conjugated secondary antibodies were obtained from Dako. Chemiluminescent detection was carried out using ECL Prime (GE Healthcare). Membranes were imaged using the Gel Doc XR+ system (Bio-Rad).

### Monocyte Adhesion Assay

Following density gradient separation, peripheral blood mononuclear cells from a healthy donor were harvested and monocytes were isolated using human CD14 MicroBeads UltraPure (130-118-906; Miltenyi Biotec, Germany), according to the manufacturer’s instructions.^25^ CellTracker Red CMTPX (C34552; Invitrogen) at a concentration of 2 µM was used to fluorescently label the monocytes, according to the manufacturer’s instructions. ECs were cultured under flow using the orbital shaker model as described above and stimulated with TNF (10 ng/mL) for the last 4 hours of flow. The media was removed and replaced with media containing fluorescently labeled monocytes (1×10^6^ per well) and the cells were incubated for 2 hours under static conditions. The wells were washed gently with PBS to remove nonadherent monocytes and fixed in paraformaldehyde (4% w/v). DAPI (Sigma) was used to label the nuclei. Fluorescent images were taken using the ×20 objective of a wide-field microscope (DM14000B; Leica) to detect adherent monocytes. An average of 6 images were analyzed per well and used to calculate the average number of adherent monocytes per EC per sample.

### In Silico Analysis of Predicted Transcription Factor Binding Sites

We used UCSC (University of California, Santa Cruz) genome browser (https://genome.ucsc.edu/) and human assembly GRCh38/hg38 to view the predicted binding sites for transcription factor profiles in the JASPAR CORE collection.^26^

### Zebrafish Studies

All zebrafish studies conformed to the institution’s ethical requirements, UK Home Office regulations, and guidelines from Directive 2010/63/EU of the European Parliament on the protection of animals used for scientific purposes. Zebrafish care and experimental procedures were carried out under Project Licence 70/8588 issued by the UK Home Office. Maintenance, manipulation, and staging of transgenic line *flk1:EGFP-NLS*^27^ were carried out as described in standard husbandry protocols.^28,29^ ECs were isolated by fluorescence-activated cell sorting from dissociated *flk1:EGFP-NLS* embryos, as described previously.^30^ Morpholino antisense oligonucleotides (MOs; GeneTools, LLC) were diluted in sterile water and ≈1 nL was injected into the yolk of a 1- to 4-cell stage embryo. Nontargeting control MO was used as a negative control. Final concentration of 3 ng of *eva1a* and control MO was used for the experiments. The list of MOs used in this study is presented in Table S2. The efficiency of *eva1a* splice-blocking MO was validated by reverse transcriptase-PCR (RT-PCR) and qRT-PCR. Total RNA was extracted from zebrafish embryos using RNeasy Mini Kit (Qiagen) according to manufacturer’s protocol and 500 ng of total RNA was subjected to cDNA synthesis using iScript reverse transcriptase (Bio-Rad). Resulting cDNA was used as a template for PCR using gene-specific primers (listed in Table S1) and BioMix Red kit (Bioline) as per manufacturer’s instructions. The resulting RT-PCR products were analyzed by agarose gel electrophoresis. Amplification of housekeeping gene beta-actin (*bact2*) was used as an internal control. Apoptotic cells in whole-mount zebrafish embryos were detected by active caspase 3 staining, as previously described.^31^ Imaging was performed using an Olympus FV1000 laser scanning confocal microscope with a ×40 (numerical aperture 1.0) oil immersion objective. EC apoptosis was quantified by counting the total number of ECs (GFP^+^ [green fluorescent protein-positive] cells; green) and the number of apoptotic ECs (GFP^+^ and active caspase 3^+^; yellow). The proportion of apoptotic cells was obtained by dividing the number of apoptotic cells by the total number of cells.

### Statistical Analysis

Data are presented as means values±SEM. Statistical analyses were performed with GraphPad Prism software. The test performed is indicated in the figure legend and *P* values are shown in the graphs. Data have been tested for normality and equal variance using the Shapiro-Wilk and Brown-Forsythe tests, respectively. For sample size n≤5 per group a nonparametric statistical test was performed.

## Results

### Proatherogenic Shear Stress Induces EVA1A Expression in Pigs, Mice, and Human ECs

**Figure 1.**
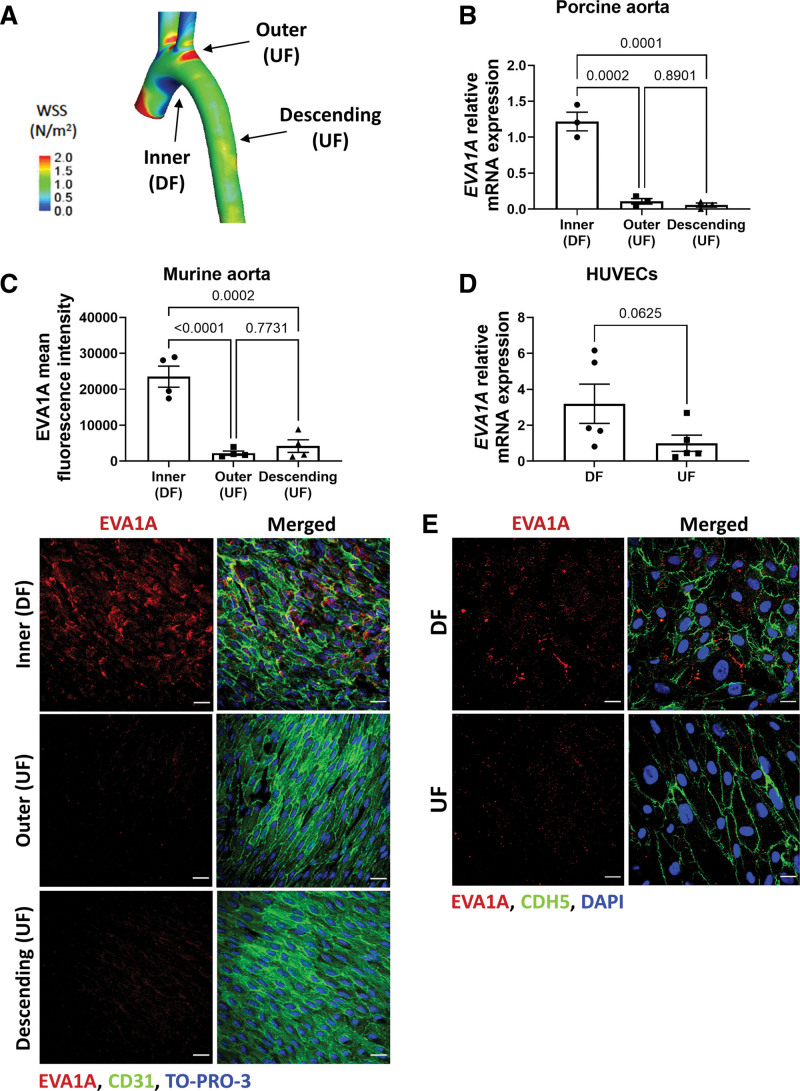
**EVA1A (eva-1 homolog A) is induced by proatherogenic shear stress in pig, mouse, and human endothelial cells (ECs). A**, Map of time-averaged wall shear stress in the porcine aorta. The regions of undisturbed flow (UF) and disturbed flow (DF) are indicated by arrows. Adapted from Serbanovic-Canic et al.^32^ **B**, ECs were isolated from the inner curvature of the porcine aortic arch (DF site), outer curvature of the aortic arch (UF site), and the descending aorta (UF site). The mRNA expression of *EVA1A* was quantified in each cell population by quantitative real-time polymerase chain reaction (qRT-PCR; n=3 pigs) and normalized to housekeeping gene *B2M*. **C**, Aortas were isolated from 8-wk-old C57BL/6 mice (n=4 mice) and en face immunostaining was performed using anti-EVA1A antibody (**C**, red) in the inner and outer curvature of the aortic arch and the descending aorta. The endothelium was stained with anti-CD31 antibody (**C**, green) and nuclei costained with TO-PRO-3 (**C**, blue). The analyzed data was from a minimum of 3 fields of view per animal per aortic location. The graph in **C** represents EVA1A mean fluorescence intensity (n=4 mice). **D** and **E**, Human umbilical vein EC (HUVECs) were cultured under flow for 72 h using the orbital shaker model. **D**, Cells were isolated from DF (center of the well) and UF (periphery of the well) regions and *EVA1A* mRNA expression levels were measured by qRT-PCR (n=5 donors). *EVA1A* expression was normalized to housekeeping gene *HPRT*. **E**, Protein levels and cellular localization of EVA1A were assessed by immunostaining using anti-EVA1A antibody (red), EC marker cadherin 5 (CDH5, green), and costaining of nuclei (DAPI [4′,6-diamidino-2-phenylindole], blue; n=3 donors). **B, C**, and **D**, Data are presented as means±SE of the mean. Differences between groups were analyzed using 1-way ANOVA with Tukey posthoc test (**B** and **C**) and Wilcoxon sum rank test (**D**). *P* values are shown in the graphs. Scale bar: (**C**), 40 µm; (**D**), 20 µm.

The effect of flow on the expression of EVA1A was studied in the aortas of healthy pigs and mice (Figure 1A through 1C). EVA1A showed increased mRNA expression (pig aorta, Figure 1B) and protein expression (mouse aorta, Figure 1C) in the inner curvature of the aortic arch (DF region) compared to the outer curvature and the descending aorta (UF regions). EVA1A protein localization was mainly in the plasma membrane and cytoplasmic, showing a vesicular pattern of expression (Figure S1), in accordance with its known intracellular localization.^8,33^

Next, EVA1A expression was studied in human umbilical vein ECs (HUVECs) and human aortic ECs (HAECs) exposed to flow using the orbital shaker model which generates DF in the center of the well and UF in the periphery of the well.^21,34^ EVA1A mRNA and protein expression levels were increased by DF (Figure 1D and 1E and Figure S2) and EVA1A immunostaining showed comparable cellular EVA1A localization to that observed in the mouse aorta. Taken together, these data indicate that expression of EVA1A is regulated by WSS and is induced by the proatherogenic DF both in vivo and in vitro.

### EVA1A Induces Apoptosis and Increases Permeability and Inflammatory Activation of Human ECs Under Proatherogenic Shear Stress

**Figure 2.**
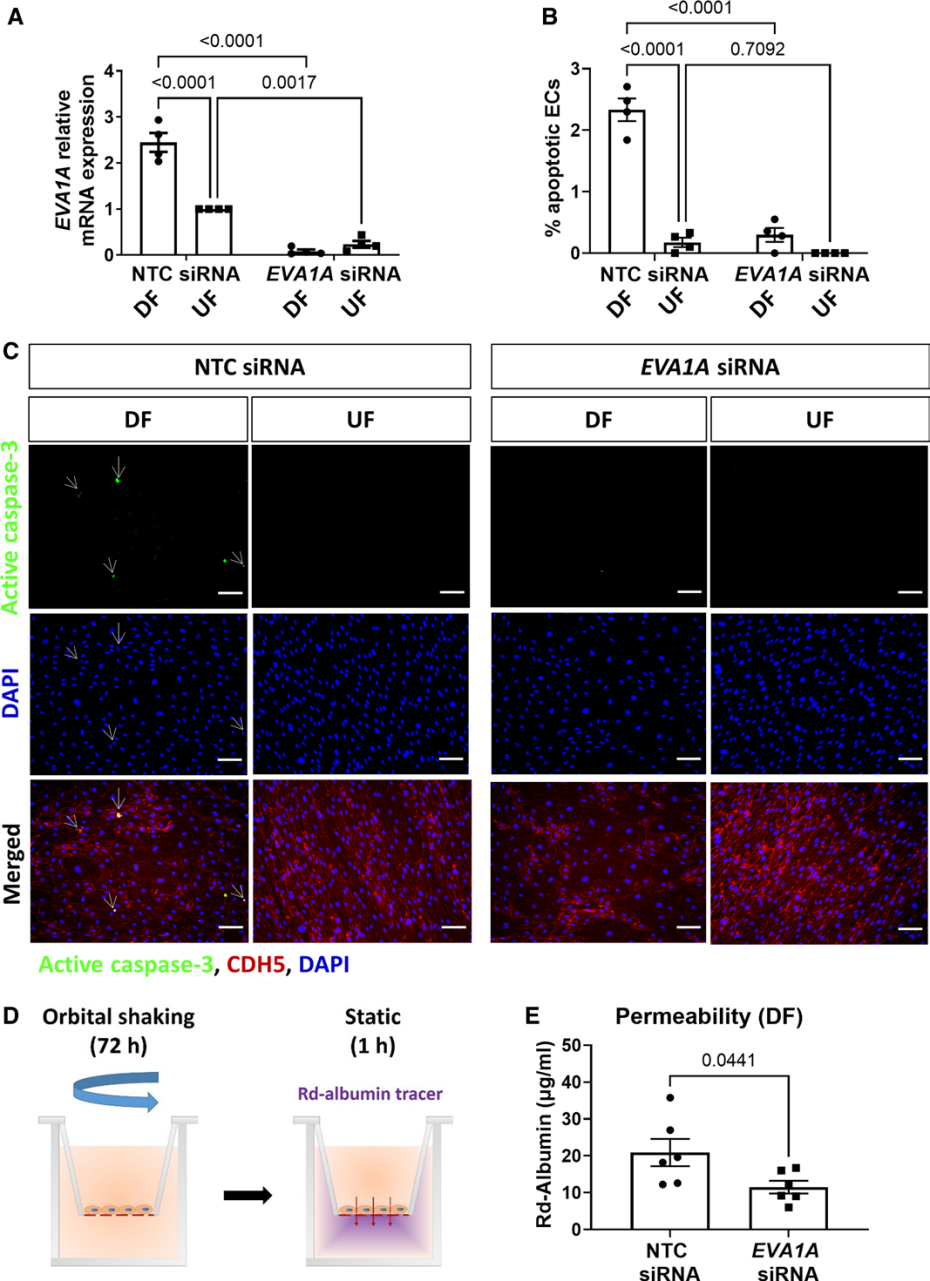
**EVA1A (eva-1 homolog A) promotes endothelial apoptosis under proatherogenic disturbed flow. A** through **C**, Human umbilical vein endothelial cells (HUVECs) were treated with *EVA1A* siRNA (small interfering RNA) or with nontargeting control (NTC) siRNA before exposing to flow for 72 h using the orbital shaker system. **A**, ECs were isolated from disturbed flow (DF) and undisturbed flow (UF) regions and the efficiency of *EVA1A* siRNA was assessed by quantitative real-time polymerase chain reaction (qRT-PCR; n=4 donors) using *HPRT* as a housekeeping gene. *EVA1A* relative expression was normalized to UF NTC siRNA. **B** and **C**, EC apoptosis under DF and UF was assessed by immunostaining using antiactive caspase 3 antibody (**C**, green) and costaining with EC marker CDH5 (cadherin 5; **C**, red) and DAPI (4′,6-diamidino-2-phenylindole; **C**, blue). Apoptotic ECs are indicated with white arrows. The graph in **B** represents percentage of apoptotic ECs calculated by dividing the number of active caspase 3-positive cells by the total number of EC per field of view (n=4 donors). **D** and **E**, HUVECs were treated with *EVA1A* siRNA or with nontargeting control (NTC) siRNA and cultured on transwell inserts. ECs were exposed to DF for 72 h using the orbital shaker system before assessment of endothelial permeability under static conditions for 1 h using rhodamine (Rd)-albumin as a tracer. The graph in **E** represents concentration of Rd-albumin measured in the lower compartment (n=6). **A**, **B**, and **E**, Data are presented as means±SEM. Differences between groups were analyzed using a 2-way ANOVA (**A** and **B**) with Tukey post hoc test or unpaired *t* test (**E**) and *P* values are shown in the graphs. Scale bar: (**C**), 100 µm.

Proatherogenic DF induces EC dysfunction by inducing inflammatory activation and EC turnover (via increased apoptosis and proliferation), leading to increased EC permeability.^5^ To study the role of EVA1A in human ECs under physiological flow conditions, *EVA1A* was knocked down in HUVECs using siRNA. *EVA1A* siRNA reduced expression levels of *EVA1A* mRNA by ≈97% under DF conditions and ≈77% under UF conditions (Figure 2A). The effect of EVA1A knockdown on EC apoptosis was assessed by active caspase 3 staining of ECs exposed to flow for 72 hours using the orbital shaker model. In accordance with previous studies,^22,32^ ECs exposed to DF showed significantly increased levels of apoptosis (2.33±0.18% apoptotic ECs) compared with ECs exposed to UF (0.17±0.08% apoptotic ECs; Figure 2B and 2C). EVA1A deficiency reduced EC apoptotic levels under DF conditions to those comparable to UF conditions (0.30±0.11% apoptotic ECs), indicating that EVA1A promotes EC apoptosis under DF (Figure 2B and 2C). To test whether the effect of EVA1A on EC apoptosis is flow-dependent, we analyzed EC apoptosis under static conditions. Although there was a trend towards decreased apoptosis in *EVA1A*-deficient ECs (1.03±0.22% apoptotic cells) compared to control cells (1.96±0.52% apoptotic cells), this was not statistically significant (Figure S3).

In addition to EC apoptosis, dysregulated EC proliferation can also contribute to increased endothelial turnover.^35^ Therefore, we tested whether EVA1A regulates EC proliferation by analyzing the expression of proliferation marker PCNA in *EVA1A*-deficient ECs exposed to flow or under static conditions (Figure S4). No significant difference in the proportion of PCNA^+^ proliferating cells was observed when EVA1A was knocked down compared to control cells under any of the studied conditions (Figure S4).

**Figure 3.**
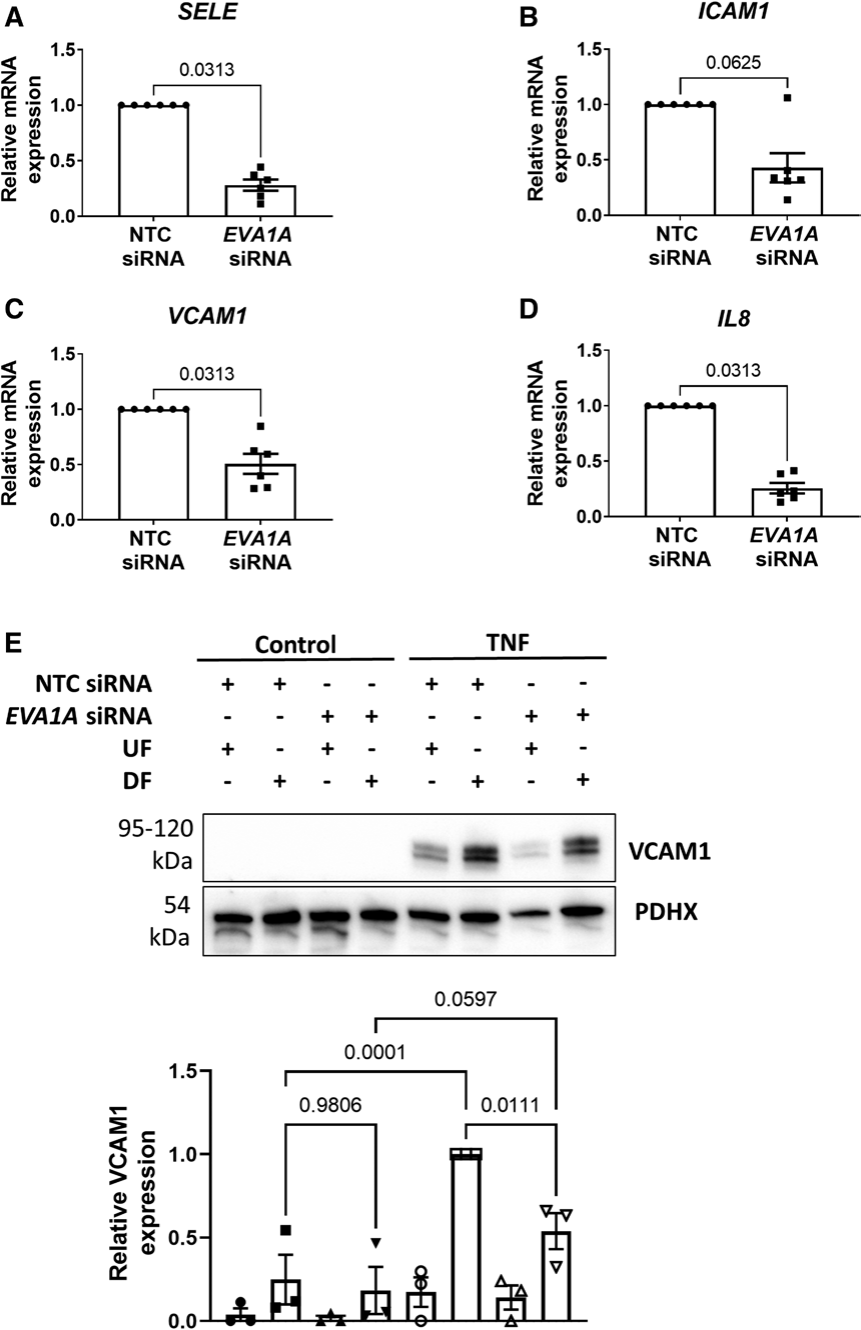
**EVA1A (eva-1 homolog A) promotes endothelial permeability and inflammatory activation under disturbed flow. A** through **E**, Human umbilical vein endothelial cells (HUVECs) were treated with *EVA1A* siRNA (small interfering RNA) or with nontargeting control (NTC) siRNA before exposing to flow for 72 h using the orbital shaker system. **A** through **D**, ECs were isolated from the disturbed flow (DF) region, and mRNA expression of E-selectin (*SELE*), intercellular adhesion molecule 1 (*ICAM1*), vascular cell adhesion molecule (*VCAM1*), and interleukin 8 (*IL8*) were measured by quantitative real-time polymerase chain reaction (qRT-PCR; n=6), using *HPRT* as a housekeeping gene. **E**, ECs were stimulated with TNF (tumor necrosis factor; 10 ng/mL) for the last 4 h of flow. VCAM1 protein expression was assessed in TNF-stimulated and nonstimulated (control) ECs isolated from the DF and undisturbed flow (UF) regions by Western blotting. Graph shows VCAM1 protein levels normalized to PDHX (pyruvate dehydrogenase complex component X; n=3). **A** through **E**, Data are presented as means±SEM and normalized to control. Differences between groups were analyzed using a nonparametric Wilcoxon test (**A–D**) or a 2-way ANOVA with Tukey post hoc test (**E**). *P* values are shown in the graphs.

Since apoptosis has been implicated in vascular leakiness,^36,37^ we assessed the effect of EVA1A knockdown on EC permeability using the Transwell assay (Figure 2D and 2E and Figure S5). Following 72 hours of exposure to DF, *EVA1A*-deficient ECs were less permeable to a fluorescent tracer, Rd-Albumin (rhodamine-labeled albumin; 11.5±1.7 µg/mL), compared to control cells (20.9±3.7 µg/mL Rd-Albumin) after 1 hour incubation, (Figure 2E). At the same time, *EVA1A* knockdown had no significant effect on permeability under static conditions (12.2±2.5 μg/mL) compared with control cells (13.0±1.3 μg/mL; Figure S5). To better understand the role of EVA1A in regulating EC dysfunction, we measured the expression of several inflammatory cytokines and adhesion molecules induced by DF in *EVA1A*-deficient ECs. Knockdown of *EVA1A* resulted in a significant decrease of mRNA levels of E-selectin (*SELE*), *VCAM1*, and interleukin 8 (*IL8*) under DF conditions, whereas there was a nonsignificant trend for decreased expression of *ICAM1* (Figure 3A through 3D). However, under UF or static conditions, EVA1A deficiency did not lead to a change in inflammatory marker expression (Figure S6), indicating that the effect of EVA1A on inflammatory activation is dependent on flow conditions.

To test whether increased mRNA levels of inflammatory markers match that of the protein, we measured VCAM1 protein expression by Western blotting (Figure 3E). In nonstimulated cells, VCAM1 protein levels were low in both *EVA1A*-deficient and control cells. Upon stimulation with TNF (tumor necrosis factor), there was a significant upregulation of VCAM1 expression under DF compared with UF conditions in control cells, while knockdown of *EVA1A* rescued this effect of TNF on DF-induced VCAM1 upregulation (Figure 3E). To examine functional consequences of reduced inflammatory activation in the absence of EVA1A under DF conditions, we performed a monocyte adhesion assay. Knockdown of *EVA1A* in TNF-stimulated ECs resulted in a trend towards decreased adhesion of monocytes to the endothelium under DF, which, however, did not reach statistical significance (Figure S7), indicating that EVA1A may be priming ECs for monocyte adhesion.

In summary, these data suggest that EVA1A mediates the effects of DF on EC apoptosis and inflammatory activation, leading to increased EC permeability.

### EVA1A Promotes EC Dysfunction by Regulating Autophagic Flux in ECs Exposed to DF

**Figure 4.**
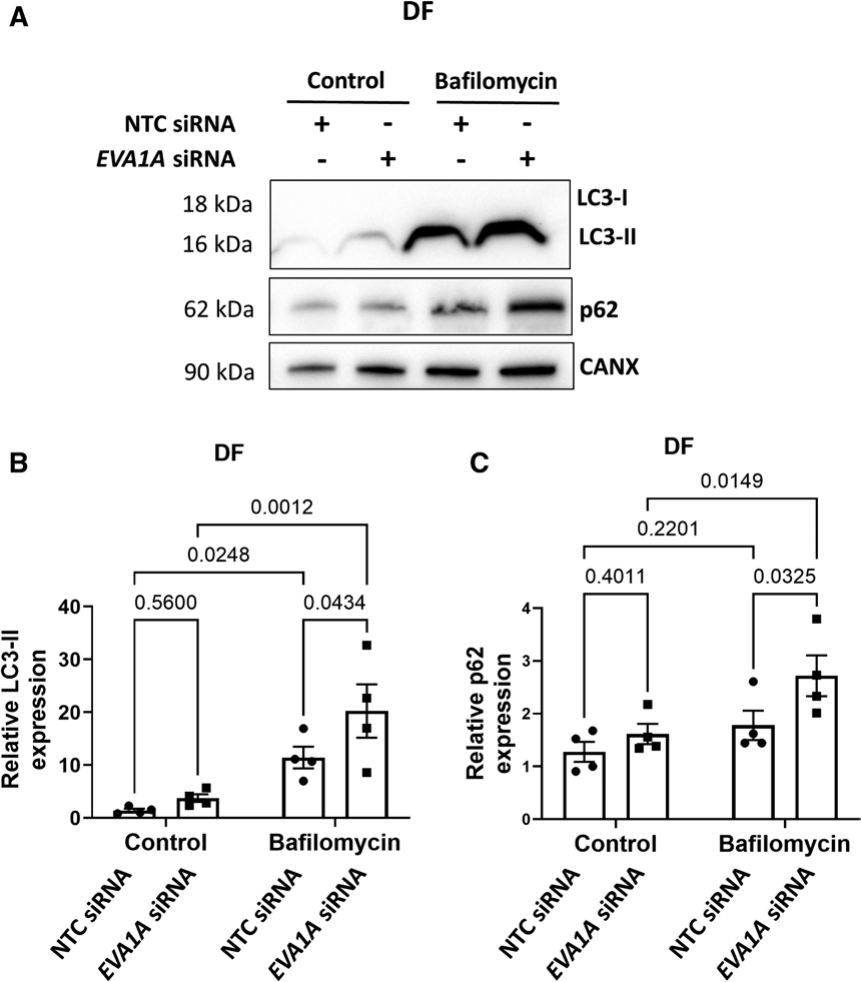
**EVA1A reduces autophagic flux under disturbed flow. A** through **C**, Human umbilical vein endothelial cells (HUVECs) were treated with *EVA1A* siRNA (small interfering RNA) or with nontargeting control (NTC) siRNA before exposing them to flow for 72 h using the orbital shaker system. To block autophagic flux, ECs were treated with 50 nM bafilomycin for the last 4 h of flow exposure. Control cells were treated with 0.05% dimethyl sulfoxide (DMSO). ECs were isolated from the disturbed flow (DF) region and expression levels of autophagy markers LC3-II (microtubule-associated protein 1 light chain 3-II) and p62 were assessed by Western blotting. Calnexin was used as a loading control. **B** and **C**, Graphs show protein levels of LC3-II and p62 normalized to CANX (calnexin; n=4). Data are presented as means±SE of the mean. Differences between groups were analyzed using a 2-way ANOVA (**B** and **C**) with Tukey post hoc test and *P* values are shown in the graphs.

EVA1A is a regulator of apoptosis and autophagy in several different cell types, during both physiological and pathological processes.^38^ To test whether EVA1A plays a role in autophagy regulation in ECs under physiological flow conditions, we measured autophagic flux in *EVA1A*-silenced ECs by using autolysosome inhibitor, bafilomycin. Bafilomycin blocks autophagic flux and prevents degradation of autophagy markers LC3-II and p62, allowing the distinction between true autophagy induction and blockade of autophagic flux.^39^ The difference in the amount of LC3-II and p62 in the presence and absence of bafilomycin, which is a measure of autophagic flux, was observed in control and *EVA1A*-silenced cells exposed to flow (Figure 4A through 4C and Figure S8). In the absence of bafilomycin, upon exposure to DF, there was a trend toward increased LC3-II expression in *EVA1A*-deficient cells compared to controls, which was not significant (Figures 4A and 4B). However, blocking autophagic flux using bafilomycin led to a significant enrichment in LC3-II (77.1% increase) and p62 (52.7% increase) protein levels in *EVA1A*-silenced cells compared with control ECs, indicating an increase in autophagic flux in cells lacking EVA1A (Figure 4A through 4C). In contrast, under UF conditions, there was no difference in LC3-II and p62 levels between *EVA1A*-knockdown cells and controls either in the presence or in the absence of bafilomycin (Figure S8), suggesting that EVA1A does not modulate autophagy under UF conditions. The LC3-I form of LC3 was virtually undetectable by Western blotting, suggesting that, under the studied conditions, the majority of LC3 is in the form of autophagy-associated LC3-II, rather than cytoplasmic form, LC3-I (Figure S9). This is consistent with previous reports looking at the effects of chronic flow exposure on autophagy markers in ECs.^40^

**Figure 5.**
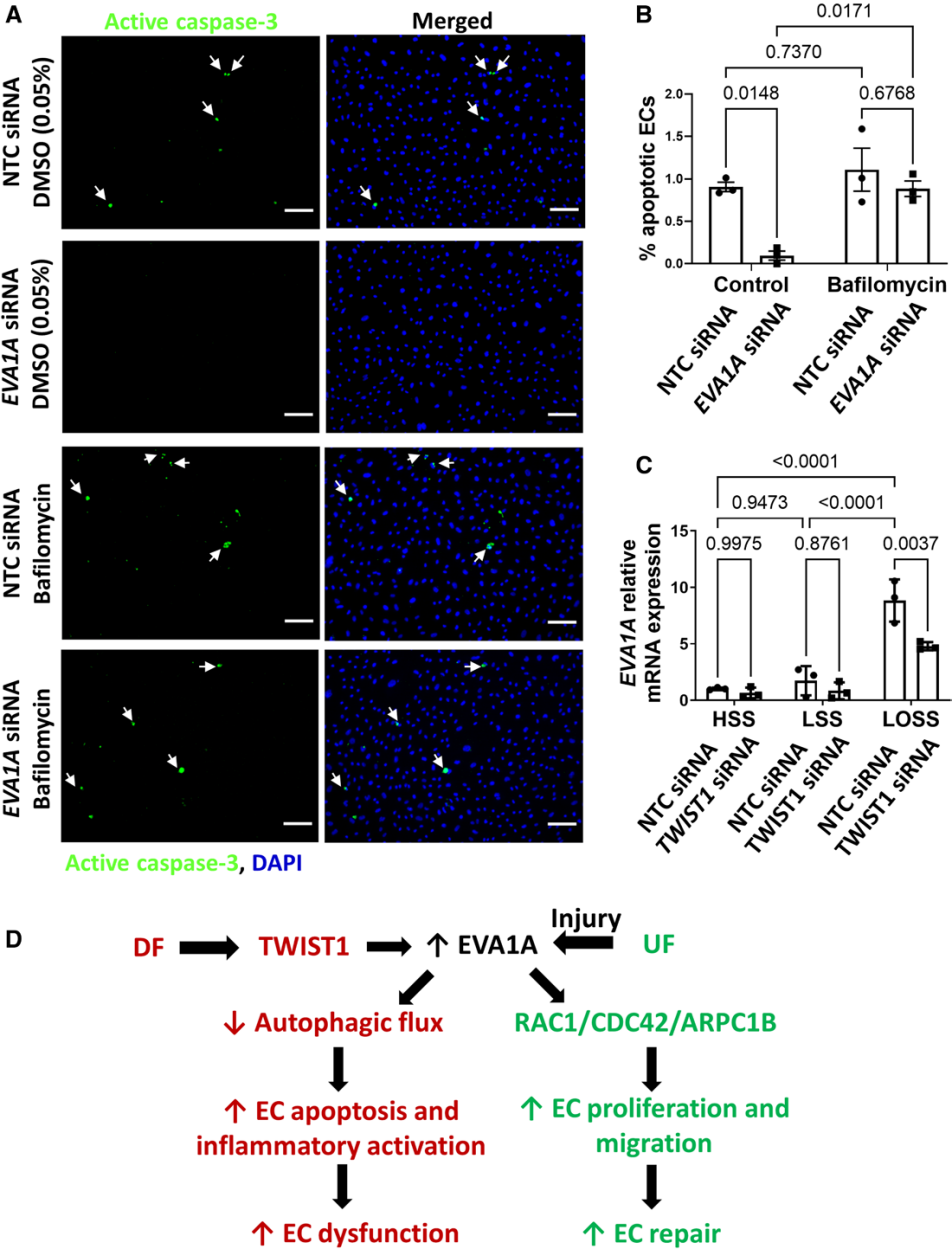
**EVA1A (eva-1 homolog A) promotes endothelial apoptosis via regulation of autophagy. A** and **B**, Human umbilical vein endothelial cells (HUVECs) were treated with *EVA1A* siRNA (small interfering RNA) or with nontargeting control (NTC) siRNA before exposing to flow for 72 h using the orbital shaker system. To block autophagic flux, ECs were treated with 50 nM bafilomycin for the last 4 h of flow exposure. Control cells were treated with 0.05% dimethyl sulfoxide (DMSO). EC apoptosis under disturbed flow (DF) was assessed by immunostaining using antiactive caspase 3 antibody (**A**, green) and costaining of nuclei with DAPI (4′,6-diamidino-2-phenylindole; **A**, blue). Apoptotic ECs are indicated with white arrows. The graph in **B** represents percentage of apoptotic ECs calculated by dividing the number of active caspase 3–positive cells by the total number of ECs per field of view (n=3). **C**, Human aortic ECs (HAECs) were treated with *TWIST1* (twist basic helix-loop-helix transcription factor 1) siRNA or with NTC siRNA before exposing to flow for 72 h using the ibidi parallel plate system. The ECs were exposed to either high laminar shear stress (HSS) of 13 dynes/cm^2^, low laminar shear stress (LSS) of 4 dynes/cm^2^, or low oscillatory shear stress (LOSS) of 4 dynes/cm^2^ with 1 Hz oscillations. Expression of *EVA1A* mRNA was measured by quantitative real-time polymerase chain reaction (qRT-PCR; n=3 donors), using *HPRT* as a housekeeping gene. **B** and **C**, Data are presented as means±SE of the mean and normalized to control (**C**). Differences between groups were analyzed using a 2-way ANOVA with Tukey post hoc test and *P* values are shown in the graphs. **D**, A schematic diagram summarizing proposed mechanism of how EVA1A (*Continued* )**Figure 5 Continued.** regulates endothelial function under different hemodynamic conditions. Under DF conditions, EVA1A expression is upregulated in ECs and contributes to the DF-induced reduction in autophagic flux, leading to increased EC apoptosis and inflammatory activation, which results in EC dysfunction and lesion development (this study). EVA1A expression can also be induced in injured ECs under undisturbed flow (UF) conditions, where it promotes EC repair by regulating EC proliferation and migration via RAC1/CDC42/ARPC1B (rac family small GTPase 1/cell division cycle 42/actin-related protein 2/3 complex subunit 1B) pathway (study by Li et al^20^). Scale bar: (**A**), 100 µm.

To determine whether EVA1A promotes EC apoptosis under DF by regulating autophagy, we assessed the effect of blocking autophagic flux on EC apoptosis in EVA1A-silenced cells (Figures 5A and 5B). *EVA1A*-silencing in control cells (treated with 0.05% dimethyl sulfoxide [DMSO]) showed a significant decrease in EC apoptosis (0.10±0.05% apoptotic ECs) compared to control cells (0.90±0.05% apoptotic ECs), consistent with the proapoptotic function of EVA1A. However, bafilomycin treatment rescued apoptosis in *EVA1A*-deficient cells, bringing it to levels comparable to control cells (0.88±0.09% apoptotic ECs; Figure 5A and 5B). At the same time, treatment of DF-exposed control cells with bafilomycin per se did not result in a significant increase in EC apoptosis (1.11±0.25% apoptotic ECs).

We conclude that EVA1A reduces autophagic flux in ECs exposed to proatherogenic DF, leading to EC dysfunction.

### EVA1A Expression Is Regulated by Flow Direction and Transcription Factor TWIST1

Atherogenic DF is characterized by both low WSS magnitude and changes in WSS direction. To dissect which of these 2 components of DF is responsible for EVA1A induction in ECs, we used a parallel plate flow system (Figure 5C). Interestingly, *EVA1A* mRNA expression was not induced when ECs were exposed to laminar low WSS (4 dynes/cm^2^) compared with high laminar WSS (13 dynes/cm^2^) but was significantly upregulated (≈8-fold) when ECs were exposed to low oscillatory WSS (LOSS) of 4 dynes/cm^2^ oscillating at 1 Hz. Therefore, our data suggest that it is the disturbance in flow direction, rather than WSS magnitude, that leads to EVA1A upregulation when ECs are exposed to DF conditions.

We were next interested in better understanding the mechanism underlying the mechanosensitive regulation of EVA1A. In silico investigation of predicted transcription factor binding sites revealed several putative binding sites for transcription factor TWIST1 in the 5’ upstream region within 1 kb from the transcription start site, as well as in the first exon and intron of EVA1A gene (Figure S10). The identified sites are located in the close vicinity of the ENCODE candidate cis-Regulatory elements classified as a promoter-like signature (marked in red in Figure S10), proximal enhancer-like signatures (marked in orange in Figure S10), and distal enhancer-like signatures (marked in yellow in Figure S10), therefore increasing the likelihood that these predicted TWIST1 binding sites are functional. TWIST1 is a basic helix-loop-helix transcription factor that is activated by DF and promotes endothelial dysfunction and atherosclerosis,^32,41,42^ making it an attractive candidate for the DF-sensitive regulation of EVA1A. We tested whether TWIST1 regulates *EVA1A* mRNA expression by knocking down TWIST1 in ECs using siRNA (Figure 5C). We found that TWIST1 deficiency significantly reduced *EVA1A* mRNA expression under DF conditions, suggesting that EVA1A is a downstream target of TWIST1 (Figure 5D).

### EVA1A Regulates EC Apoptosis In Vivo in Zebrafish Embryos

To test the role of EVA1A in ECs in vivo, we used our recently established zebrafish model of flow-regulated EC apoptosis.^32^ Two *EVA1A* orthologues were identified in the zebrafish genome, *eva1a-1* and *eva1a-2* (Ensembl IDs: ENSDARG00000061405 and ENSDARG00000067927, respectively), which have comparable similarity to its human EVA1A homolog, namely 66.7% and 69.3% identity at amino acid level, respectively. We first assessed whether *eva1a-1* and *eva1a-2* are expressed in zebrafish ECs. To this aim, we isolated GFP^+^ ECs from 30-hour-old *flk1:EGFP-NLS* embryos using fluorescence-activated cell sorting and analyzed gene expression using qRT-PCR (Figure S11). FACSorted GFP^+^ cells showed a significant enrichment in the expression of EC marker VE-cadherin (*cdh5*) compared to GFP^-^ cells, confirming that GFP^+^ cells are indeed endothelial (Figure S11B). *Eva1a-1* was not expressed in endothelial GFP^+^ cells, whereas *eva1a-2* was present in both endothelial GFP^+^ and nonendothelial GFP^-^ cells, showing a higher expression (≈5-fold) in the latter (Figure S11C). Therefore, we focused on *eva1a-2* orthologue to study its role in the zebrafish endothelium.

Gene-specific morpholino oligonucleotide (MO) was designed to specifically knock down *eva1a-2*, referred to as *eva1a* hereafter while having no complementary target in *eva1a-1* (Table S2). The ability of *eva1a* MO to prevent normal splicing of *eva1a* pre-mRNA at tested doses (3 and 6 ng) was confirmed by RT-PCR followed by agarose gel electrophoresis. This showed a decrease in the expression of the wild-type transcript and presence of an additional transcript corresponding to deletion of the targeted exon in *eva1a* MO-injected embryos (Figure S12A). Additionally, we confirmed that *eva1a* MO decreases *eva1a* mRNA expression levels by 83% (3 ng) and 94% (6 ng) by qRT-PCR (Figure S12B), validating the efficiency of *eva1a* MO.

**Figure 6.**
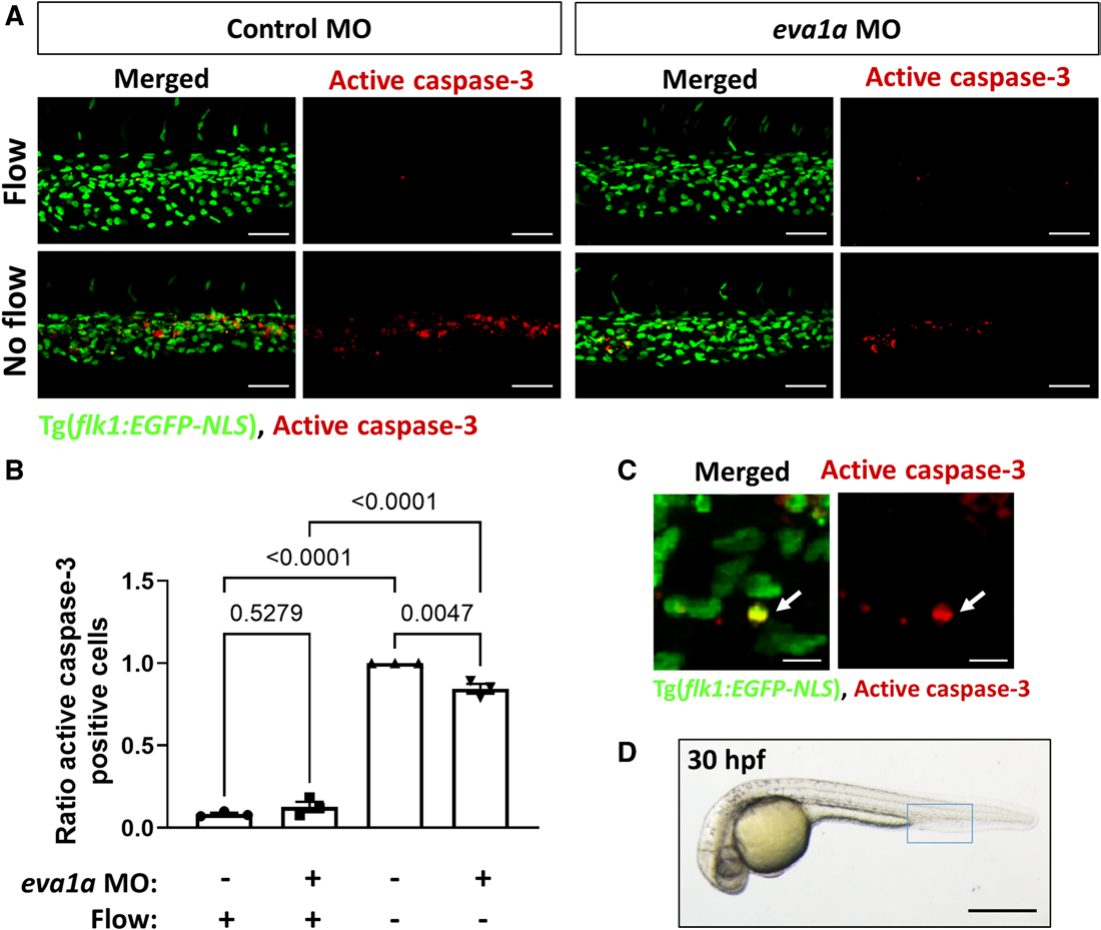
**EVA1A (eva-1 homolog A) promotes endothelial apoptosis in vivo in zebrafish. A**, Zebrafish embryos (transgenic *flk1:EGFP-NLS* embryos; green endothelial cell [EC] nuclei) were injected with morpholino oligonucleotide (MO) targeting *eva1a* or a nontargeting control MO. EC apoptosis was studied in the presence (control MO, flow) or in the absence (*silent heart* [*sih*] MO, no flow) of flow by whole-mount active caspase 3 staining (red). Apoptotic ECs (yellow) were monitored at 30 h postfertilisation (hpf). Lateral view, anterior to the **left**, dorsal up. **B**, The proportion of apoptotic ECs (number of apoptotic ECs divided by the total number of ECs) normalized to *sih* MO-injected embryos was calculated, and mean values are shown with SEM. Each data point represents an independent experiment with n≥5 embryos. Differences between groups were analyzed using a 2-way ANOVA with Tukey post hoc test and *P* values are shown in the graph. **C**, A high magnification image of EC apoptosis in a *sih* MO-injected embryo; white arrow indicates an apoptotic EC (yellow). **D**, Zebrafish embryo at 30 hpf. The region outlined with blue box represents the region that is studied in **A**. Scale bars: (**A**), 50 µm; (**C**), 10 µm; (**D**), 500 µm.

To study the effect of *eva1a* on flow-regulated EC apoptosis, blood circulation was prevented by injecting the silent heart (*sih*) MO (Figure 6). EC apoptosis was significantly enhanced by the cessation of flow (Figure 6), as shown previously.^32^ Knockdown of *eva1a* had no significant effect on the basal levels of EC apoptosis in the embryos with normal blood flow at 30 hours post fertilization, whereas it decreased EC apoptosis induced by the absence of flow by 15% (Figure 6A and 6B). At the same time, there was no significant difference in the total number of ECs observed in each group at 30 hours post fertilization (Figure S13), indicating that the decrease in EC apoptosis observed in *eva1a*-deficient embryos is not a consequence of altered EC numbers. Therefore, EVA1A promotes EC apoptosis in vivo in zebrafish embryos in the absence, but not the presence, of blood flow.

## Discussion

Responses of ECs to flow include activation of complex networks of signaling pathways which play key roles in maintaining cellular quiescence or promoting EC dysfunction and subsequent atherosclerotic lesion formation. ECs are able to sense and respond to mechanical forces exerted by blood flow thanks to the presence of mechanoreceptors on their surface, which convert mechanical forces into physiological responses.^43^ Several different proteins and cellular structures have been proposed to function as EC mechanoreceptors, of which the best characterized is the trimolecular complex consisting of PECAM1 (platelet EC adhesion molecule 1), VE (vascular endothelial)-cadherin and VEGFR2 (vascular endothelial growth factor receptor 2).^44^ However, it is still unclear how ECs can distinguish between different types of flow and which mechanoreceptors (or combinations of mechanoreceptors) are responsible for this. Different –omics approaches have been used to identify differentially expressed molecules at sites of lesion protection and predilection, as they may provide crucial insights into the mechanisms of cardiovascular disease development.^5,45,46^ One such shear-responsive molecule is EVA1A, which our previous transcriptomic analysis identified as a gene upregulated in the athero-prone region in the porcine aortic arch.^32^ In the present study, we further demonstrate that EVA1A is upregulated at mRNA and protein level by proatherogenic DF in pig and mouse aortas, as well as human ECs. Functionally, under DF conditions mimicking those in human arteries, EVA1A promoted apoptosis of human ECs and increased EC permeability and inflammatory activation. Therefore, EVA1A mediates the effects of DF on EC dysfunction, which precedes the development of atherosclerotic plaques. Mechanistically, we show that in ECs exposed to DF, but not UF, EVA1A contributes to the reduction of autophagic flux, which results in increased apoptosis of ECs. Finally, we demonstrate that EVA1A is induced by changes in the direction, rather than magnitude of WSS, and identify proatherogenic transcription factor TWIST1 as an upstream regulator of EVA1A.

EVA1A regulates autophagy in different cell types, including cancer cells, hepatocytes, neural cells, and cardiomyocytes.^10–12,16–18,33,47^ The proposed mechanism involves interaction of EVA1A C-terminus with autophagy-related (ATG) ATG16L1 and recruitment of ATG12-5/ATG16L1 complex to the autophagic membrane.^48^ In cancer cells, EVA1A-mediated increase in autophagy led to increased cell death due to apoptosis.^10–12,47,48^ Autophagy plays an important role in maintaining cellular homeostasis; however, it can be a double-edged sword, having a dual role in cytoprotection and cell death, depending on the cellular context, microenvironment, and stage of disease.^49^ In the endothelium, autophagy levels are regulated by WSS and this has implications for the development of atherosclerosis.^50^ Autophagy is required for EC alignment under physiological UF and EC-specific deletion of *Atg5* in atherosclerotic mice resulted in increased lesions in the descending aorta, which is a UF site.^40^ However, autophagy is impaired under DF, leading to increased EC mitochondrial DNA damage, apoptosis, and inflammation.^40,51^ We found that DF in our in vitro model resulted in ≈2.5% apoptotic cells after 72 hours of flow exposure, which was rescued to almost non-detectable levels of apoptosis when EVA1A was knocked down. This relatively low rate of apoptosis corresponds to the in vivo situation^22^ and represents only a snapshot in time, as apoptotic cells detach from neighboring cells. Therefore, this level of interruption of the endothelial monolayer integrity is likely to have biological consequences in vivo. Endothelial barrier function is disrupted in early atherosclerosis, leading to deposition of molecules such as low-density lipoprotein, and cells in the intima.^52^ The mechanism by which EVA1A increases EC permeability remains to be elucidated. We observed no difference in EC proliferation rate in *EVA1A*-deficient cells, suggesting that the effect of EVA1A on EC permeability may be due to the loss of cell-cell contact as a result of increased EC apoptosis or direct effect of EVA1A on cell adhesion.

Our data indicate that accumulation of EVA1A under DF leads to EC apoptosis and induction of inflammatory markers, which may be the result of the reduction of autophagic flux mediated by EVA1A.

Investigation into mechanisms underlying EVA1A shear sensitivity revealed that changes in WSS direction, rather than low WSS magnitude, induce EVA1A expression in ECs. In silico analysis of predicted transcription factor binding sites in *EVA1A* gene identified multiple binding sites for transcription factor TWIST1 close to *EVA1A* promoter. TWIST1 plays pleiotropic functions in vascular disease and development and we have previously described the role of TWIST1-mediated endothelial-to-mesenchymal-transition in atherosclerosis development.^41^ We now identify EVA1A as a novel target of TWIST1, while it remains to be determined whether TWIST1 indeed directly regulates *EVA1A* gene expression. Since *EVA1A* expression was not completely abolished in TWIST1-deficient cells, additional mechanisms may be involved. Reactive oxygen species and oxidative stress play a vital role in the DF-induced inflammatory and apoptotic responses of ECs.^53^ Additionally, there is a crosstalk between reactive oxygen species and autophagy, reactive oxygen species activate autophagy, whereas autophagy substrate p62 regulates redox signaling via KEAP1/NRF2 (kelch-like ECH-associated protein 1/nuclear factor erythroid 2-related factor 2) pathway.^54^ It will, therefore, be of interest to study the potential involvement of reactive oxygen species in EVA1A-medited EC dysfunction.

As an in vitro system to expose ECs to flow, we use the orbital shaker model which has several advantages, including the ability to expose the cells to both DF and UF profiles within the same well.^34^ Importantly, WSS metrics,^37,55,56^ such as time-averaged WSS, transverse WSS, and oscillatory shear index, have a proatherogenic profile in the center of the well (DF region), characterized by low time-averaged WSS, high time-averaged WSS, transverse WSS, and high oscillatory shear index. Conversely, at the periphery of the well (UF region), there is a high time-averaged WSS, low time-averaged WSS, transverse WSS, and low oscillatory shear index, which corresponds to an atheroprotective profile.^34^ One disadvantage of the system is the difficulty of separating different flow metrics. We took advantage of a parallel plate flow system, to be able to separate the effects of WSS magnitude and flow direction. Therefore, the 2 complementary flow systems generate different mechanical environments and the use of both systems provides invaluable insights into the shear-regulated endothelial responses.

To validate our findings in vivo, we used zebrafish embryos to study the effect of EVA1A on flow-regulated EC apoptosis.^32^ It is estimated that ≈70% of human genes have at least one obvious zebrafish orthologue and the zebrafish model is increasingly being included in studies of human diseases with the aim of providing independent verification of gene function.^57–59^ Due to whole-genome duplication that occurred early during the evolution of ray-finned fishes, many human genes have >1 orthologue in the zebrafish genome.^60^ This was the case for EVA1A, which has two zebrafish orthologues with high level of conservation with human EVA1A (≈70% identities at amino acid level). One of the orthologues was not expressed in zebrafish ECs and was therefore excluded from subsequent studies. By targeting the second orthologue, which showed abundant expression in zebrafish ECs, we observed that EVA1A promotes EC apoptosis in vivo in zebrafish embryos, indicating a conserved function between fish and mammals. Our zebrafish model uses *sih* embryos, deficient in cardiac troponin T2 (*tnnt2a*), which lack heartbeat and consequently blood circulation.^61^ Embryos lacking blood flow remain viable for up to 5 days since a sufficient supply of oxygen and nutrients is provided by diffusion.^62^ We and others have demonstrated that absence of flow in zebrafish embryos closely mimics DF conditions in mammals, as demonstrated by the presence of EC cilia,^63^ *klf2* downregulation,^64^ *cxcr4* upregulation,^65^ and increased apoptosis levels^32^ compared with normal blood flow conditions. Because of these similarities, static condition in early zebrafish embryos may be considered analogous to DF condition in mammals. Therefore, our findings from both in vivo and in vitro models strongly suggest a proapoptotic role of EVA1A under disease-prone hemodynamic conditions.

Interestingly, our findings contrast those of Li et al,^20^ who found that EVA1A plays a protective role in the endothelium by promoting vessel repair. The underlying mechanism involved EVA1A-mediated EC proliferation and migration via activation of RAC1/CDC42/ARPC1B (rac family small GTPase 1/cell division cycle 42/actin-related protein 2/3 complex subunit 1B) pathway, whereas the effect of EVA1A on autophagy was not studied. These contrasting findings may be explained by different study conditions: the in vitro study by Li et al^20^ was performed in static conditions only, whereas our study was performed under both static and physiological flow conditions. Thus, we show that EVA1A promotes EC injury exclusively at DF sites, which could contribute to atherosclerotic lesion development. Although Li et al^20^ reported increased atherosclerosis in *EVA1A* whole-body knockout mice, they did not study the function of endothelial EVA1A in vivo which would have required an EC-specific *EVA1A* knockout, due to EVA1A ubiquitous expression and contribution of multiple cell types to atherosclerosis development.^66,67^ Additionally, it remains to be shown whether the protective role of EVA1A in the endothelium involves autophagy. In contrast to its reported protective role, our data suggest that in DF regions EVA1A promotes EC injury by reducing autophagic flux. Therefore, based on the current data, we propose that EVA1A plays a dual role in the endothelium—it can promote repair of injured ECs under physiological UF conditions by regulating EC proliferation and migration, whereas under DF conditions, it promotes EC apoptosis and inflammatory activation (Figure 5D). Such dual functions which specifically depend on hemodynamic conditions have already been demonstrated for mechanosensitive molecules such as PECAM1^68,69^ and PIEZO1 (piezo-type mechanosensitive ion channel component 1).^70^

Taking into consideration its reported roles in cancer and cardiovascular disease, there is interest in targeting EVA1A as a therapeutic strategy.^38^ Our study indicates that more detailed analyses of EVA1A function are warranted to better understand its role in vascular health and disease and its potential usefulness as a therapeutic target in cardiovascular disease.

## Article Information

### Acknowledgments

The authors thank Dr Victoria Ridger and Salman Almalki from the University of Sheffield for providing us with primary human monocytes.

### Sources of Funding

This study was supported by the British Heart Foundation Programme Grants (RG/13/1/30042 and RG/19/10/34506; P.C. Evans) and a British Heart Foundation Intermediate Fellowship (FS/18/2/33221; J. Serbanovic-Canic).

### Disclosures

None.

### Supplemental Material

Figures S1–S13

Tables S1–S2

Full Unedited Gels

## Supplementary Material
