# Supplementary material for: EVA1A (Eva-1 Homolog A) Promotes Endothelial Apoptosis and Inflammatory Activation Under Disturbed Flow Via Regulation of Autophagy
Source: Arterioscler Thromb Vasc Biol. 2023 Feb 16;43(4):547–61. doi: 10.1161/ATVBAHA.122.318110 (PMC10026973; doi:10.1161/ATVBAHA.122.318110)
Supplement: Supplementary file 1 [file atv-43-547-s001.pdf]

SUPPLEMENTAL MATERIALS

EVA1A promotes endothelial apoptosis and inflammatory activation under disturbed flow via regulation of autophagy

Canham L,[1] Sendac S,[1] Diagbouga MR,[1] Wolodimeroff E,[1] Pirri D,[2] Tardajos Ayllon B,[1] Feng S,[1] Souilhol C,[3] Chico TJA,[1] Evans PC,[1^] Serbanovic-Canic J[1^]

[^] equal contribution as senior authors

[1] *Department of Infection, Immunity and Cardiovascular Disease, INSIGNEO Institute for In Silico Medicine, and the Bateson Centre, University of Sheffield, Sheffield, UK.*

[2] *National Heart and Lung Institute, Imperial College London, London, UK.*

[3] *Biomolecular Sciences Research Centre, Sheffield Hallam University, Sheffield, UK.*

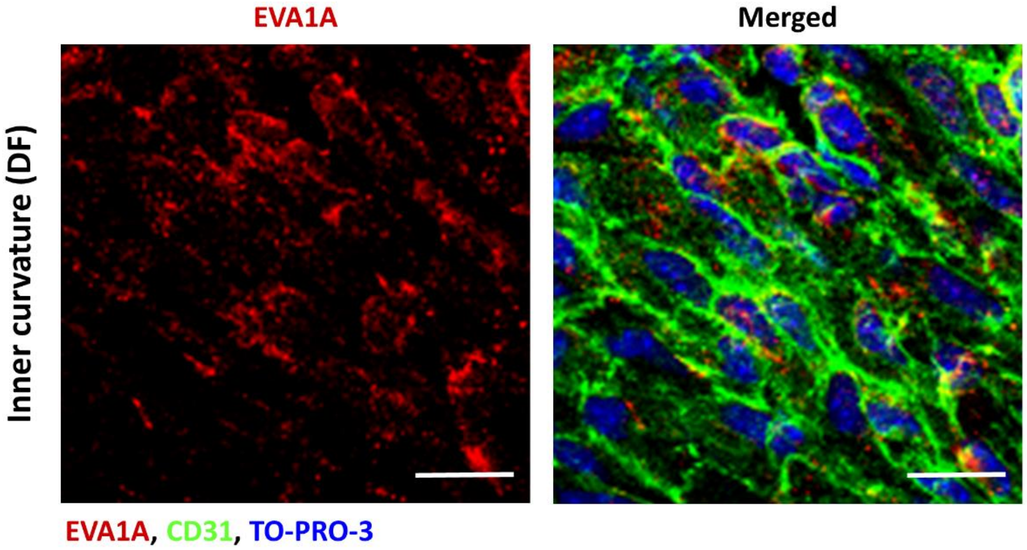

Figure S1. EVA1A is expressed in the cytoplasm and plasma membrane in the athero-prone region of mouse aorta. Higher magnification of the image shown in Figure 1C. Aortas were isolated from 8 week old C57BL/6 mice (n=4 mice) and *en face* immunostaining was performed using anti-EVA1A antibody (red) in the inner curvature of the aortic arch (disturbed flow region). The endothelium was stained with anti-CD31 antibody (green) and nuclei co-stained with TO-PRO-3 (blue). Scale bar, 40 µm.

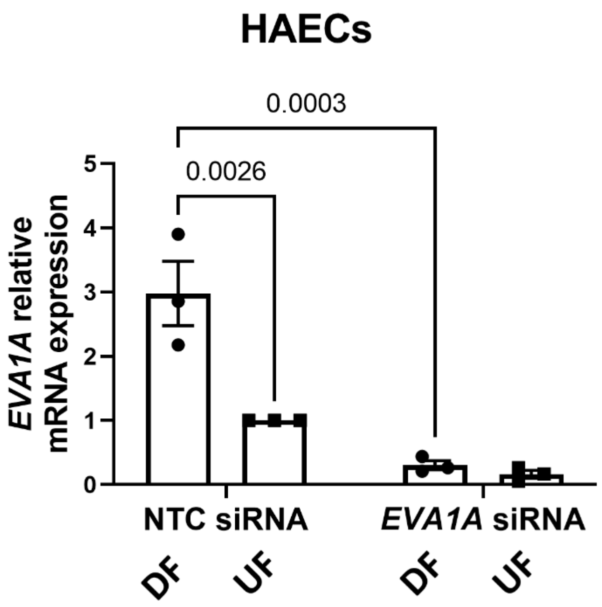

Figure S2. *EVA1A* expression in HAECs and *EVA1A* knockdown efficiency. Human aortic endothelial cells (HAECs) were treated with *EVA1A* siRNA or with non-targeting control (NTC) siRNA prior to exposing to flow for 72 hours using the orbital shaker system. ECs were isolated from disturbed flow (DF) and undisturbed flow (UF) regions and the efficiency of *EVA1A* siRNA was assessed by qRT-PCR (n=3 donors) using *HPRT* as a housekeeping gene. *EVA1A* relative expression was normalised to UF NTC siRNA. Data are presented as means ± standard error of the mean. Differences between groups were analysed using a two-way ANOVA with Tukey's post-hoc test and p-values are shown in the graph.

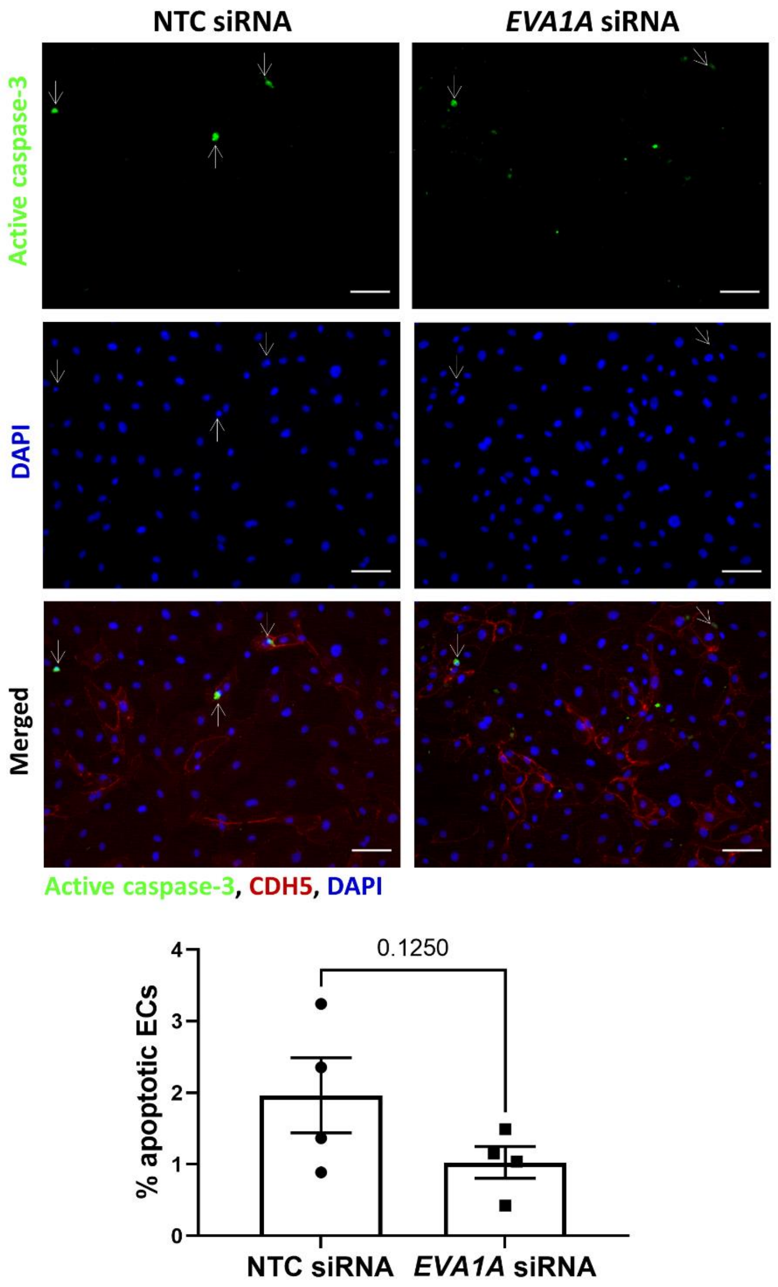

Figure S3. EVA1A has no effect on endothelial apoptosis under static conditions. HUVECs were treated with *EVA1A* siRNA or with non-targeting control (NTC) siRNA and cultured under static conditions for 72 hours. EC apoptosis was assessed by immunostaining using anti-active caspase-3 antibody (green) and co-staining with EC marker CDH5 (red) and DAPI (blue). Apoptotic ECs are indicated with white arrows. The graph represents percentage apoptotic ECs calculated by dividing the number of active caspase-3-positive cells by the total number of EC per field of view (n=4). Differences between groups were analysed using a non-parametric Wilcoxon test and p-value is shown in the graph. Scale bar: 100 µm.

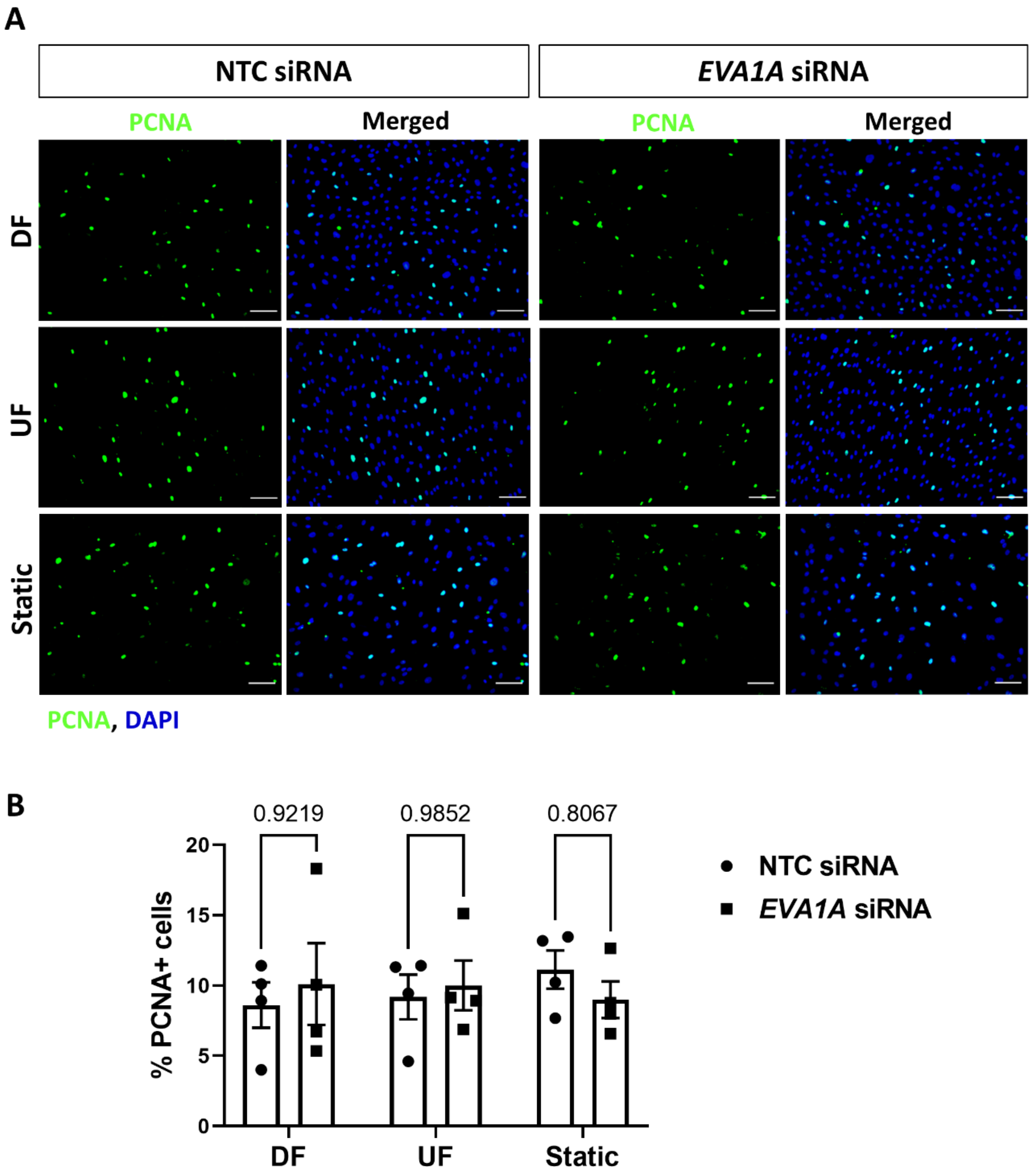

Figure S4. EVA1A has no effect on endothelial proliferation. HUVECs were treated with *EVA1A* siRNA or with non-targeting control (NTC) siRNA prior to exposing to flow for 72 hours using the orbital shaker system or were cultured in static conditions for 72 hours. (A) Proliferation of ECs under disturbed flow (DF), undisturbed flow (UF) or static conditions was assessed by immunostaining using anti-PCNA antibody (green) and co-staining of nuclei with DAPI (blue). (B) Percentage of PCNA-positive cells was calculated by dividing the number of PCNA-positive cells by the total EC number per field of view (n=4 donors). Data are presented as means ± standard error of the mean. Differences between groups were analysed using a two-way ANOVA with Sidak's post-hoc test. P-values are shown in the graph. Scale bar: (A), 100 µm.

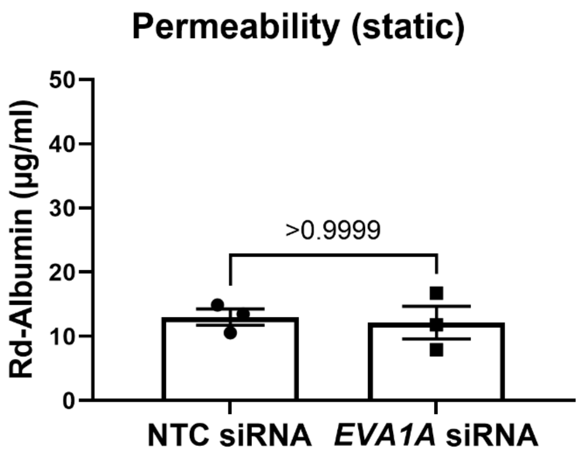

Figure S5. EVA1A has no effect on EC permeability under static conditions. HUVECs were treated with *EVA1A* siRNA or with non-targeting control (NTC) siRNA and cultured on Transwell inserts. ECs were incubated in static conditions for 72 hours prior to assessment of endothelial permeability under static conditions for 1 hour using rhodamine (Rd)-albumin as a tracer. The graph represents concentration of Rd-albumin measured in the lower compartment (n=3 donors). Differences between groups were analysed using a non-parametric Wilcoxon test and p-value is shown in the graph.

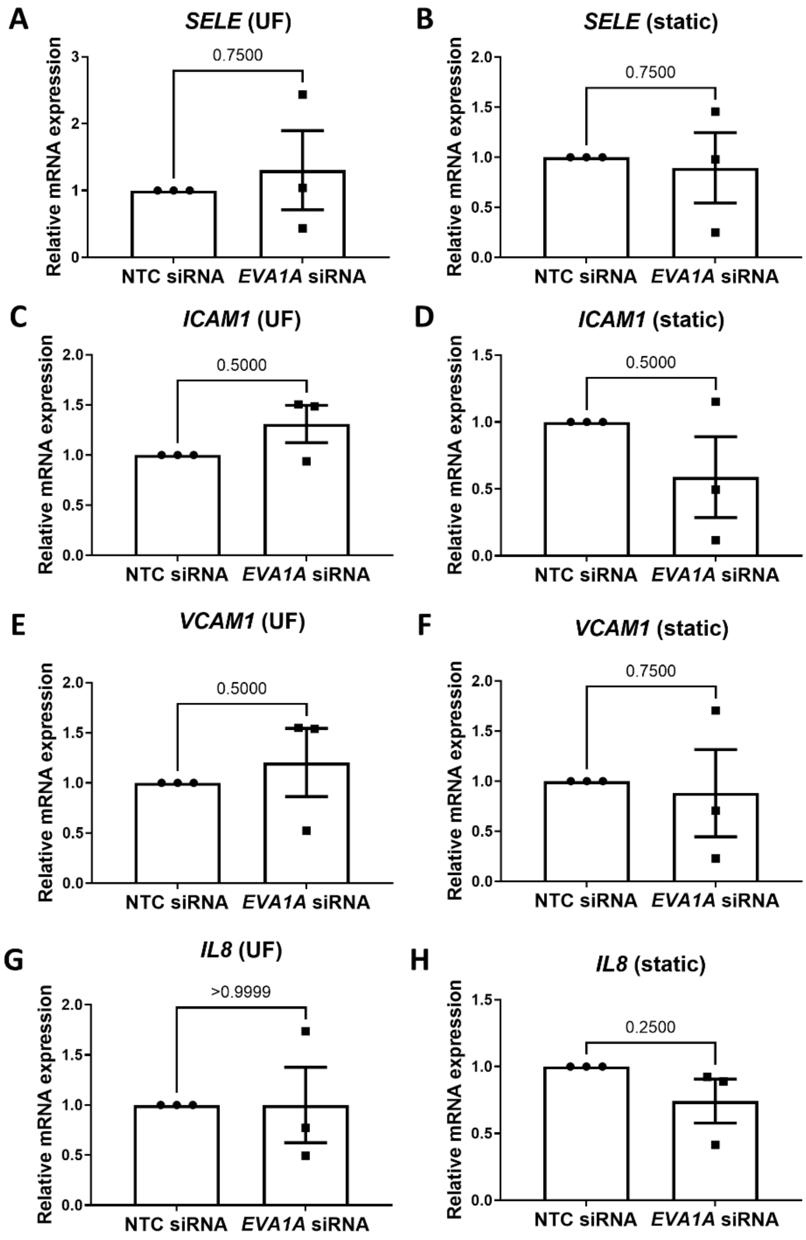

Figure S6. EVA1A has no effect on inflammatory activation under undisturbed flow or static conditions. (A-H) HUVECs were treated with *EVA1A* siRNA or with non-targeting control (NTC) siRNA prior to exposing to flow for 72 hours using the orbital shaker system or were cultured in static conditions for 72 hours. Static ECs or ECs isolated from the undisturbed flow (UF) region were used to measure mRNA expression of E-selectin (*SELE*), intercellular adhesion molecule 1 (*ICAM1*), vascular cell adhesion molecule (*VCAM1*) and interleukin 8 (*IL8*) by qRT-PCR (n=3 donors), using *HPRT* as a housekeeping gene. Data are presented as means ± standard error of the mean and normalised to control. Differences between groups were analysed using a non-parametric Wilcoxon test and p-values are shown in the graphs.

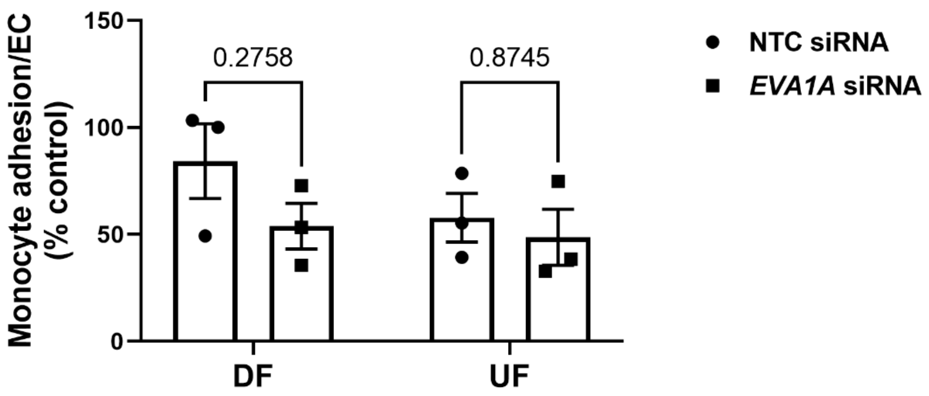

Figure S7. Effect of EVA1A knockdown on monocyte adhesion to the endothelium. Human aortic endothelial cells (HAECs) were treated with *EVA1A* siRNA or with non-targeting control (NTC) siRNA prior to exposing to flow for 72 hours using the orbital shaker system. ECs were stimulated with tumor necrosis factor (TNF) (10 ng/ml) for the last 4 hours of flow. Following the flow exposure, ECs were incubated with fluorescently labeled monocytes for 2 hours in static conditions. Adhesion of monocytes to the endothelium in the DF and UF regions was visualised and quantified as the average number of adhered monocytes per EC per field of view. Differences between groups were analysed using a two-way ANOVA with Sidak's post-hoc test and p-values are shown in the graph.

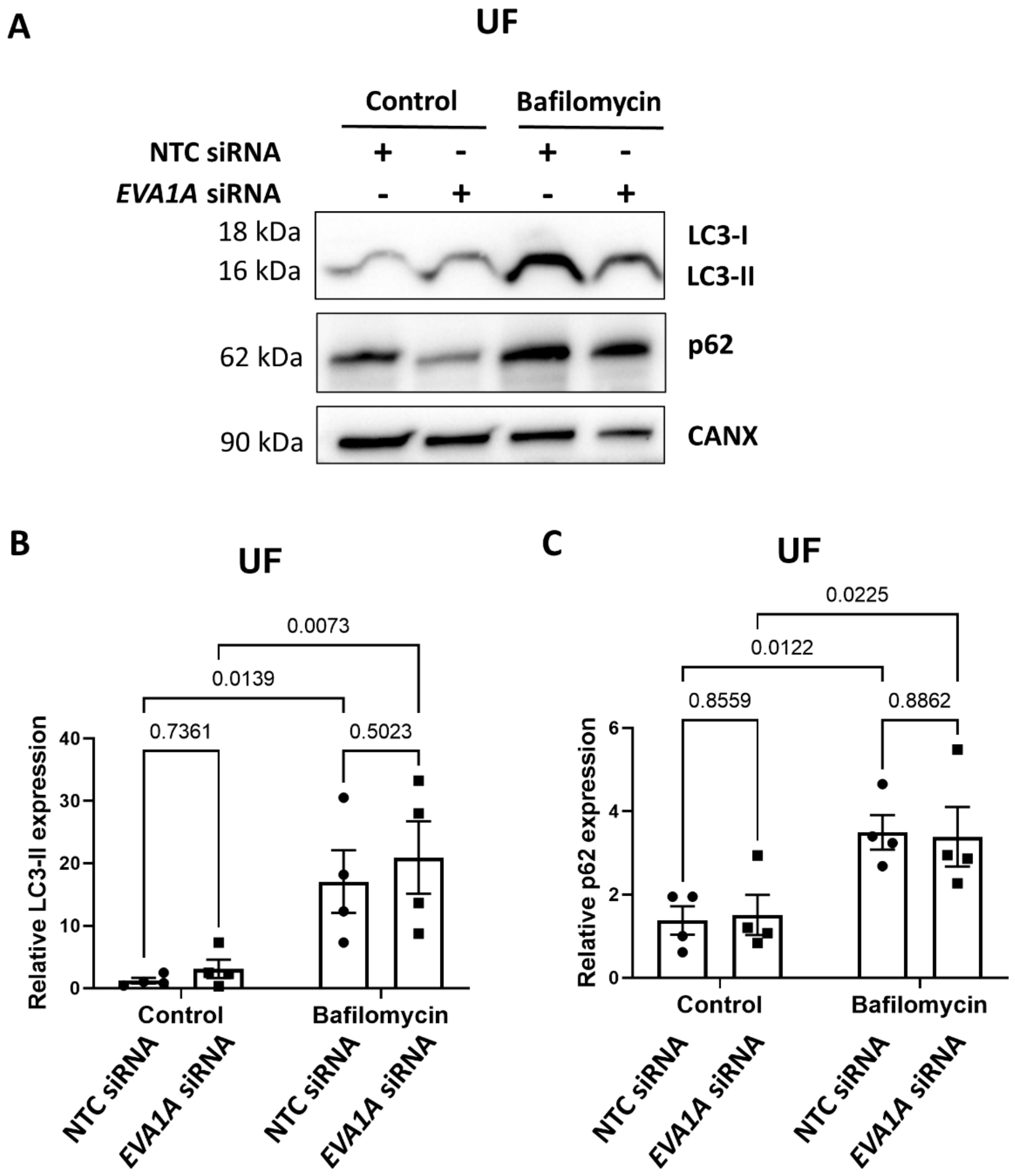

Figure S8. EVA1A has no effect on autophagic flux under undisturbed flow. (A-C) HUVECs were treated with *EVA1A* siRNA or with non-targeting control (NTC) siRNA prior to exposing to flow for 72 hours using the orbital shaker system. To block autophagic flux, ECs were treated with 50 nM bafilomycin for the last 4 hours of flow exposure. Control cells were treated with 0.05% DMSO. ECs were isolated from the undisturbed flow (UF) region and expression levels of autophagy markers LC3-II and p62 were assessed by Western blotting. Calnexin was used as a loading control. (B, C) Graphs show protein levels of LC3-II and p62 normalised to calnexin (n=4). Data are presented as means ± standard error of the mean. Differences between groups were analysed using a two-way ANOVA with Tukey's post-hoc test (B, C) and p-values are shown in the graphs.

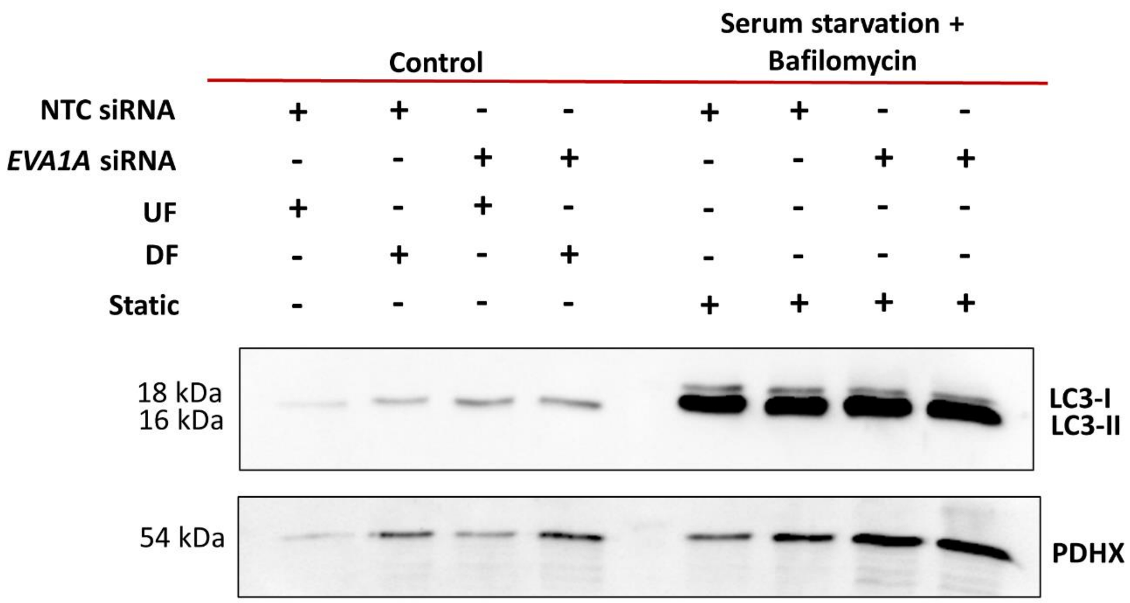

Figure S9. Control for LC3 antibody. HUVECs were treated with *EVA1A* siRNA or with non-targeting control (NTC) siRNA prior to exposing to flow for 72 hours using the orbital shaker system or were kept in static conditions for 72 hours. To stimulate autophagy, the static ECs were serum-starved for the last 24 hours of culture and to block autophagic flux, static ECs were treated with 50 nM bafilomycin for the last 4 hours. Static ECs and sheared ECs isolated from the undisturbed flow (UF) and disturbed flow (DF) regions were analysed for the expression levels of autophagy markers LC3-I and LC3-II by Western blotting. PDHX was used as a loading control.

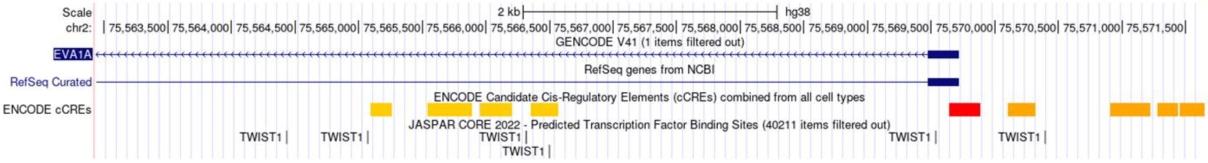

Figure S10. Predicted TWIST1 binding sites in human *EVA1A* gene. Graphic representation of predicted transcription factor TWIST1 binding sites in the vicinity of 5’ region of human *EVA1A* gene from the UCSC genome browser (https://genome.ucsc.edu/) using human assembly GRCh38/hg38. JASPAR CORE collection was used to view the predicted binding sites for TWIST1. Putative TWIST1 binding sites were identified in the 5’ upstream region within 1 kb from the transcription start site, as well as in the first exon and intron of EVA1A gene (JASPAR CORE 2022 track). The sites are located in the close vicinity of the ENCODE candidate cis-Regulatory elements (cCREs) classified as a promoter-like signature (marked in red), proximal enhancer-like signatures (marked in orange) and distal enhancer-like signature (marked in yellow) (ENCODE cCREs track).

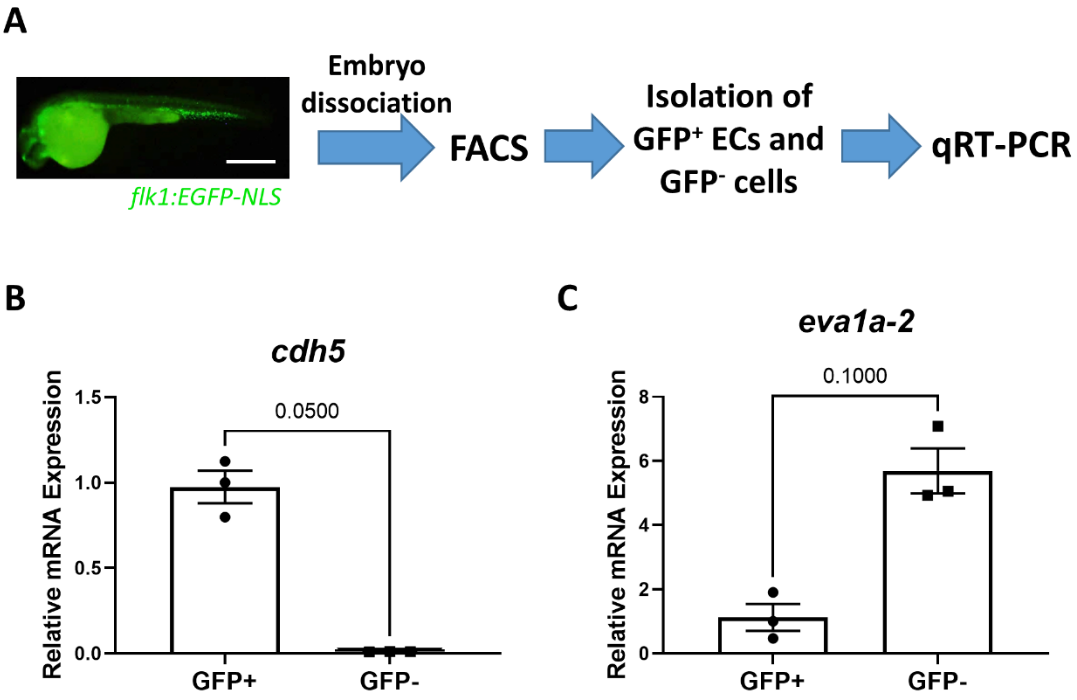

Figure S11. Assessment of endothelial *eva1a* expression in zebrafish embryos. (A-C) Endothelial cells (ECs) were isolated from Tg(*flk1:EGFP-NLS*) embryos at 30 hpf using fluorescence activated cell sorting and mRNA expression was measured in GFP^+ ECs and GFP^- non-ECs using qRT-PCR. Graphs in (B) and (C) represent relative mRNA expression of EC marker *cdh5* (B) and *eva1a* (C) in GFP^+ ECs and GFP^- cells, normalised to housekeeping gene beta actin (*bact2*). Each data point represents an independent experiment with 300-400 embryos pooled for cell sorting per experiment. Differences between groups were analysed using a non-parametric Wilcoxon test and p-values are shown in the graphs. Scale bar: (A), 500 µm.

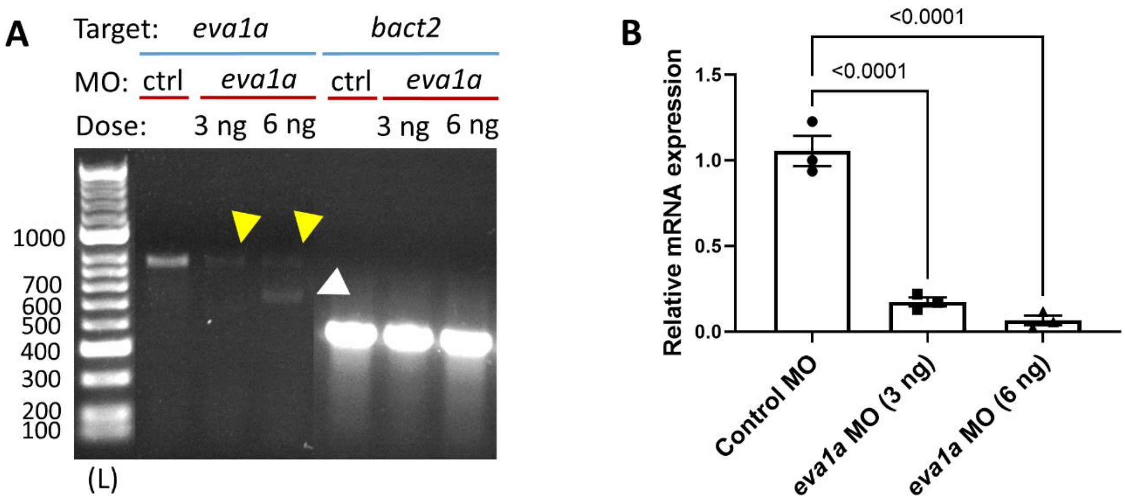

Figure S12. Validation of *eva1a* morpholino. (A-B) Zebrafish embryos were injected with *eva1a* morpholino oligonucleotide (MO) (3 ng and 6 ng) or the non-targeting control MO. Total RNA was isolated at 30 hours post fertilisation (hpf) from 15-20 embryos per group per experiment. (A) The ability of *eva1a* MO to modify splicing of the targeted transcript was assessed by analysis of RT-PCR products after agarose gel electrophoresis. Splicing modifications were observed as a band shift (indicated by white arrowhead) and as a reduction of the wildtype band (indicated by yellow arrowheads) compared to the control sample (ctrl MO). RT-PCR analysis of beta-actin (*bact2*) was used as an internal control. The DNA fragment sizes are indicated in base pairs next to the DNA ladder (L). (B) The ability of *eva1a* MO to reduce mRNA expression levels of *eva1a* was assessed by qRT-PCR, using *bact2* as a housekeeping gene. Data are presented as means ± standard error of the mean. Differences between groups were analysed using a one-way ANOVA with Tukey's post-hoc test and p-values are shown in the graph.

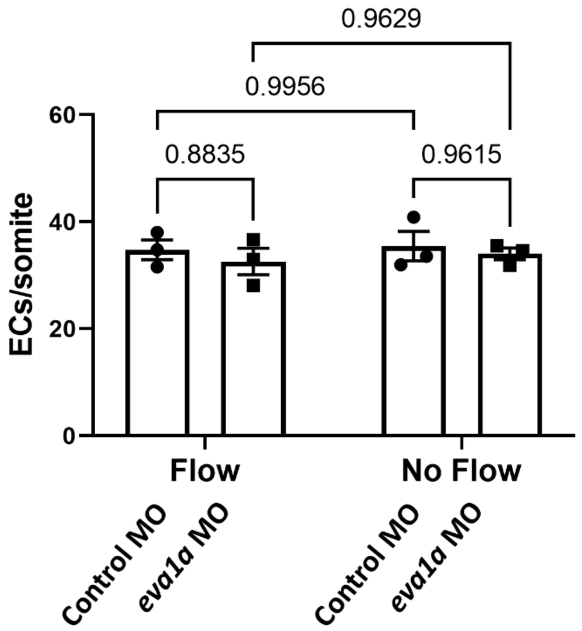

Figure S13. Eva1a knockdown in zebrafish embryos does not affect endothelial cell numbers at 30 hpf. Zebrafish embryos were injected with *eva1a* MO or the non-targeting control MO and co-injected with control MO (normal blood flow) or *sih* MO (no blood flow). The number of ECs was calculated per body segment (somite) at 30 hours post fertilisation (hpf). Each data point represents an independent experiment with ≥5 embryos pooled per experiment. Differences between groups were analysed using a two-way ANOVA with Tukey's post-hoc test and p-values are shown in the graph.

Species	Gene	Forward (5'-3')	Reverse (5'-3')	Use
Danio rerio	*bact2*	TGAAATTGCCGCACTGGTTGTTG	ATGGCGGGGGTGTTGAAGGTC	RT-PCR
Danio rerio	*bact2*	AGCCATCCTTCTTGGGTATG	TGATCTCCTTCTGCATCCTG	qRT-PCR
Danio rerio	*cdh5*	TACGCAAGGACTGGAATGAG	GGATTACGATTACCAGGATGG	qRT-PCR
Danio rerio	*eva1a-2*	AGGACTTCGCAGGACTTCTC	CCTGGAAACCGACTCCTGCA	RT-PCR
Danio rerio	*eva1a-1*	ACTGGCTGCTTACTCCTTCA	CTAGCGCGATGAGAGTGAGA	qRT-PCR
Danio rerio	*eva1a-2*	AGGTTCTCGTCAACAGTCGT	CTCTTGTACTCGTTCGGCCT	qRT-PCR
Homo sapiens	*EVA1A*	TCAGTGATCAGCTCCGGGTC	TTCTCAGACTCTTTGCTGGGG	qRT-PCR
Homo sapiens	*HPRT*	TTGGTCAGGCAGTATAATCC	GGGCATATCCTACAACAAC	qRT-PCR
Homo sapiens	*ICAM1*	AACCAGAGCCAGGAGACACT	TCTGGCTTCGTCAGAATCAC	qRT-PCR
Homo sapiens	*IL8*	TGCCAAGGAGTGCTAAG	CTCCACAACCCTCTGCAC	qRT-PCR
Homo sapiens	*SELE*	GCTCTGCAGCTCGGACAT	GAAAGTCCAGCTACCAAGGGAAT	qRT-PCR
Homo sapiens	*VCAM1*	CATTGACTTGCAGCACCACA	AGATGTGGTCCCCTCATTCG	qRT-PCR
Sus scrofa	*B2M*	TTCACTCCTAACGCTGTGGA	GTGGTCTCGATCCCACTTAAC	qRT-PCR
Sus scrofa	*EVA1A*	TCTCAGAAAATCCCGAGCGA	GAACTTCTTCTGGGGAGGCT	qRT-PCR

Table S1. PCR primers used in the study.

**Gene targeted**	**MO sequence (5'-3')**	**MO dose**	**Reference**
None – Control MO	CCTCTTACCTCAGTTACAATTTATA	variable	Gene Tools
eva1a	ATATTTCACTCTATACCTGCAAGGA	3 ng and 6 ng	This study
tnnt2a (*sih*)	CATGTTTGCTCTGATCTGACACGCA	2 ng	Sehnert et al, 2002

Table S2. Morpholinos (MOs) used in the study.

Full unedited gels for Figure 3E (1)

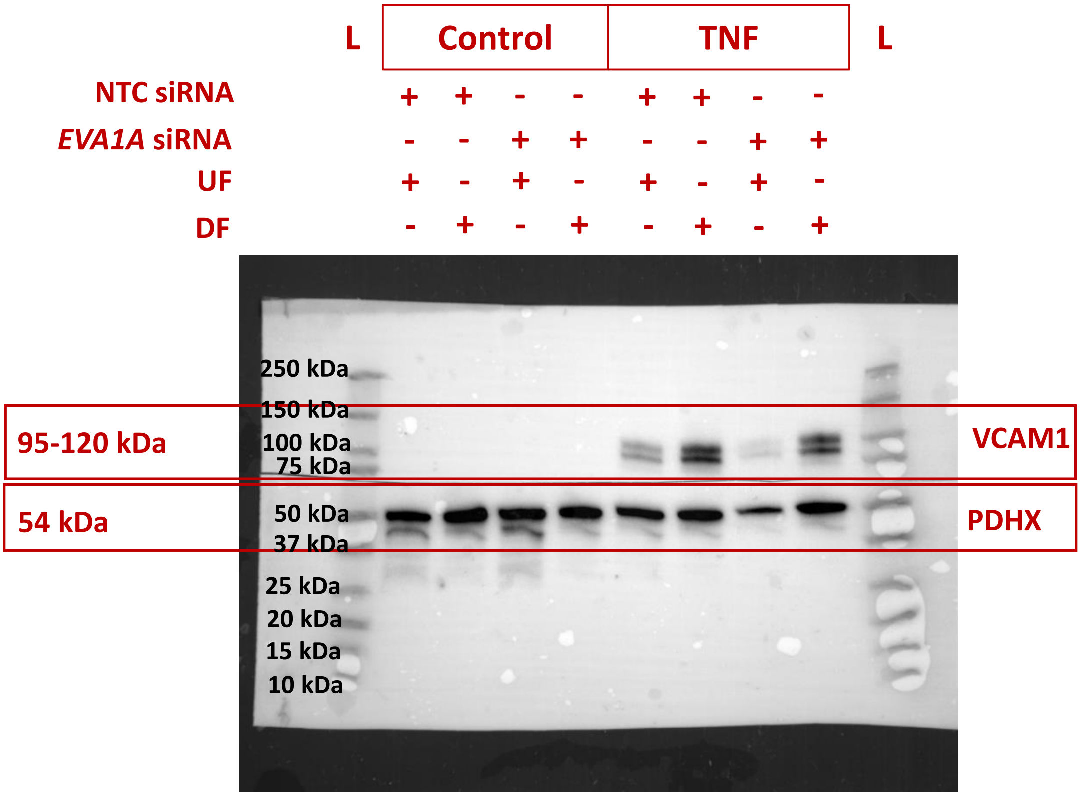

Full unedited gels for Figure 3E (2)

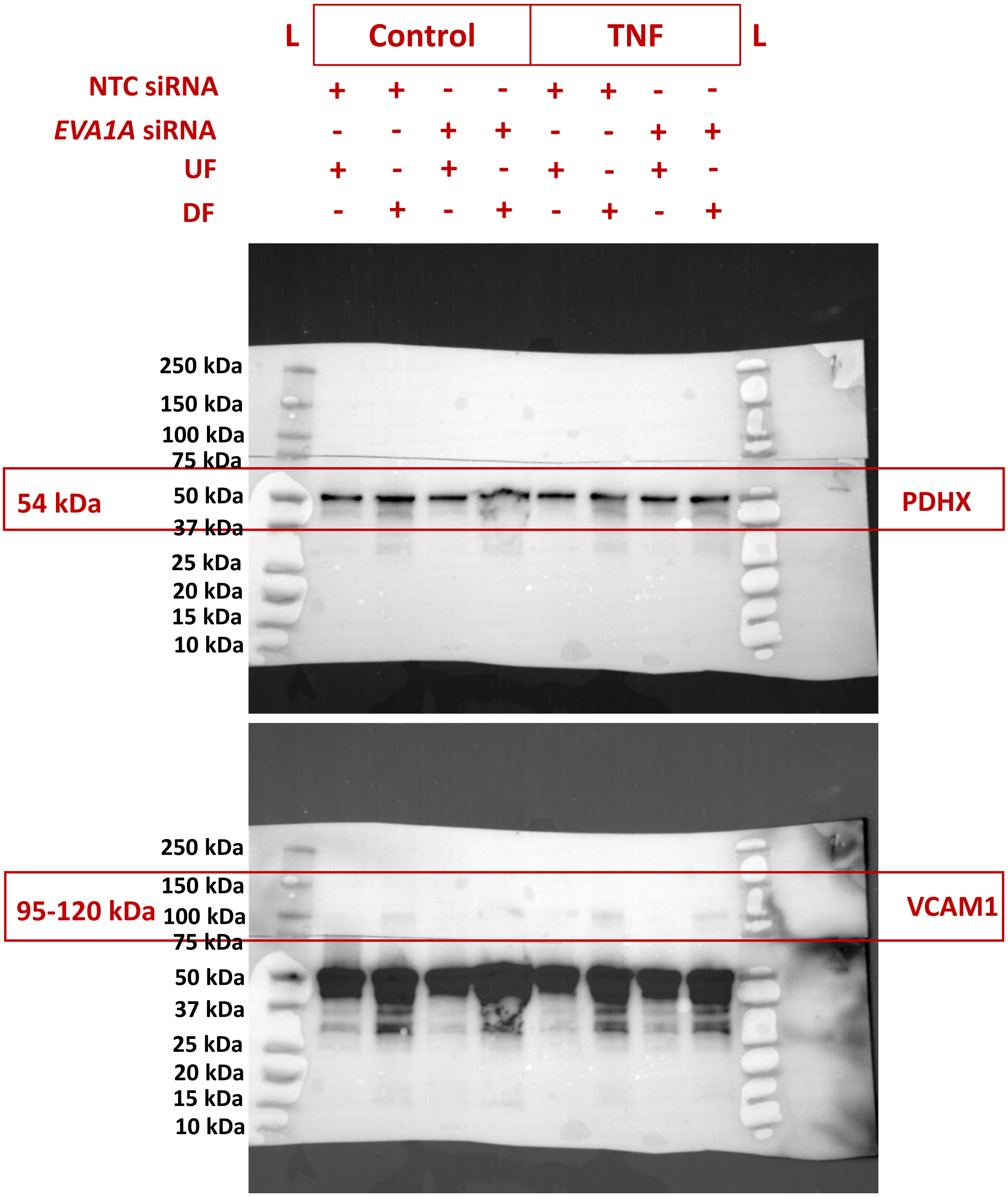

Full unedited gels for Figure 3E (3)

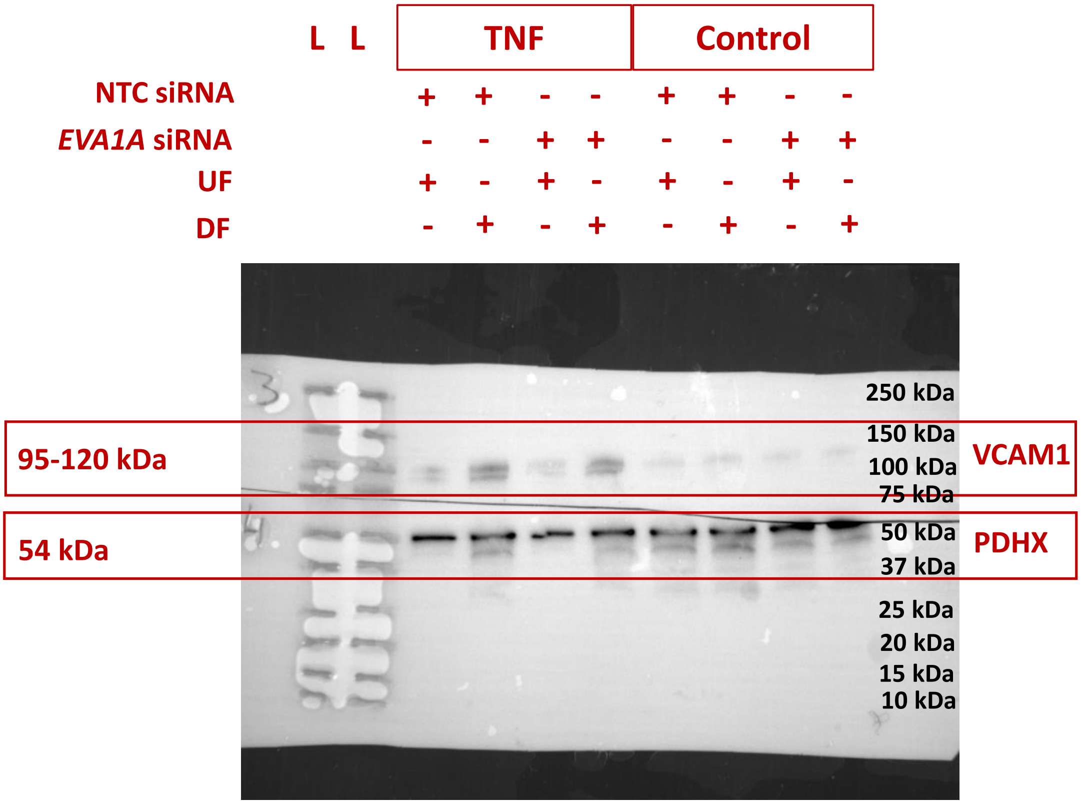

Full unedited gel for Figure 4A (1)

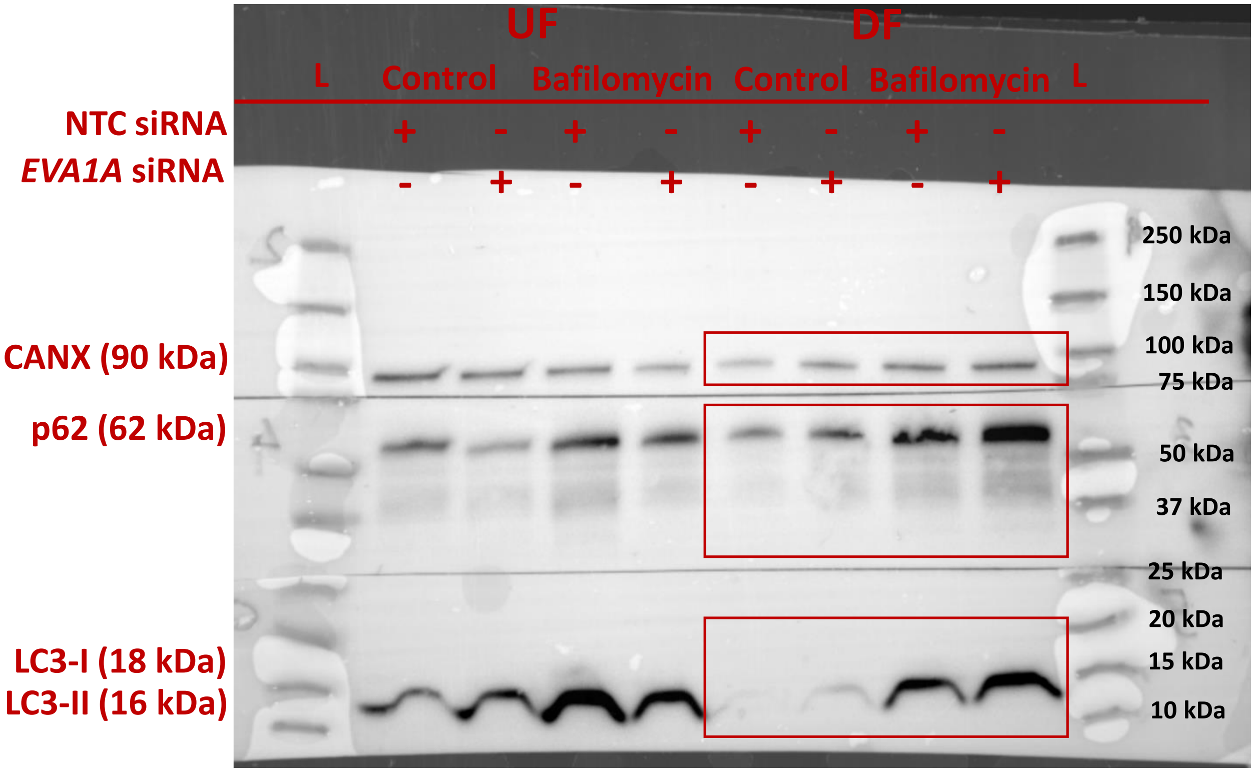

Full unedited gels for Figure 4A (2 + 3)

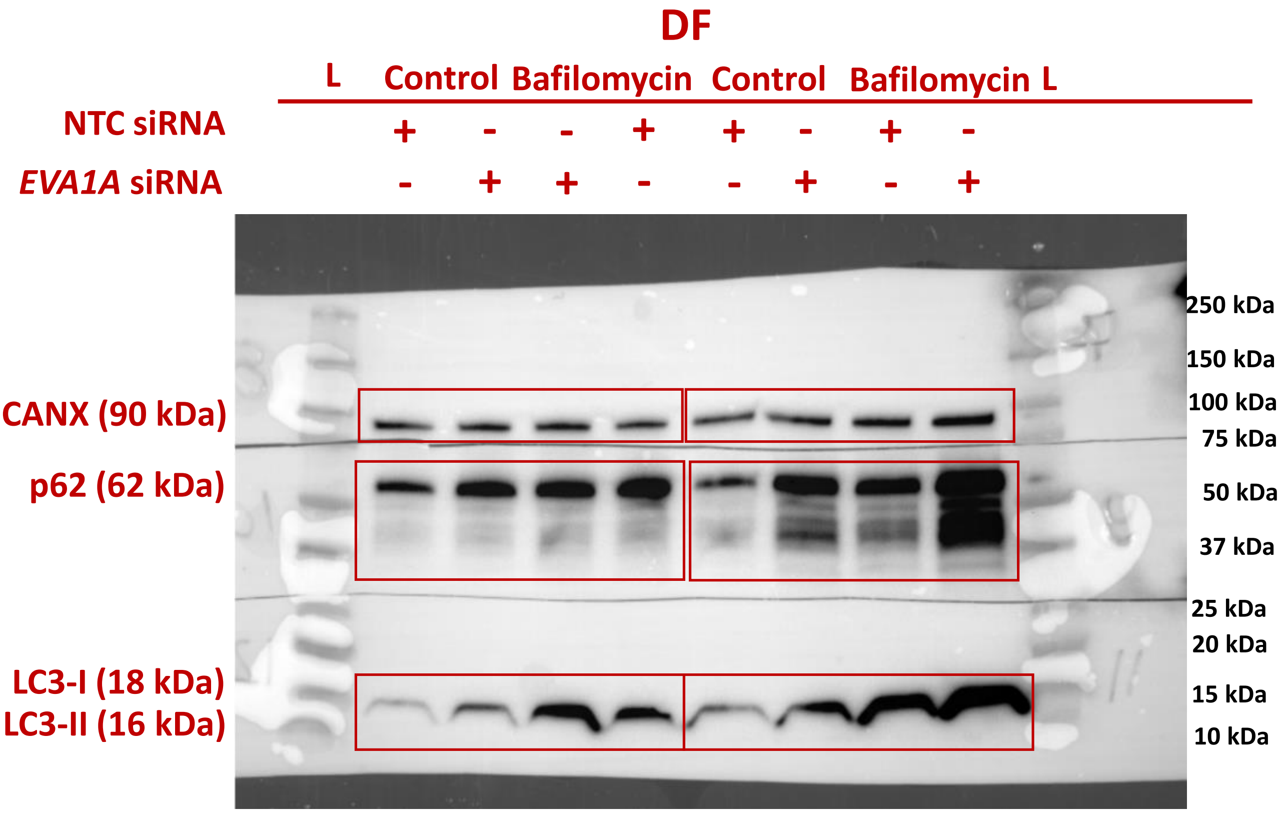

Full unedited gel for Supp. Figure 8 (1)

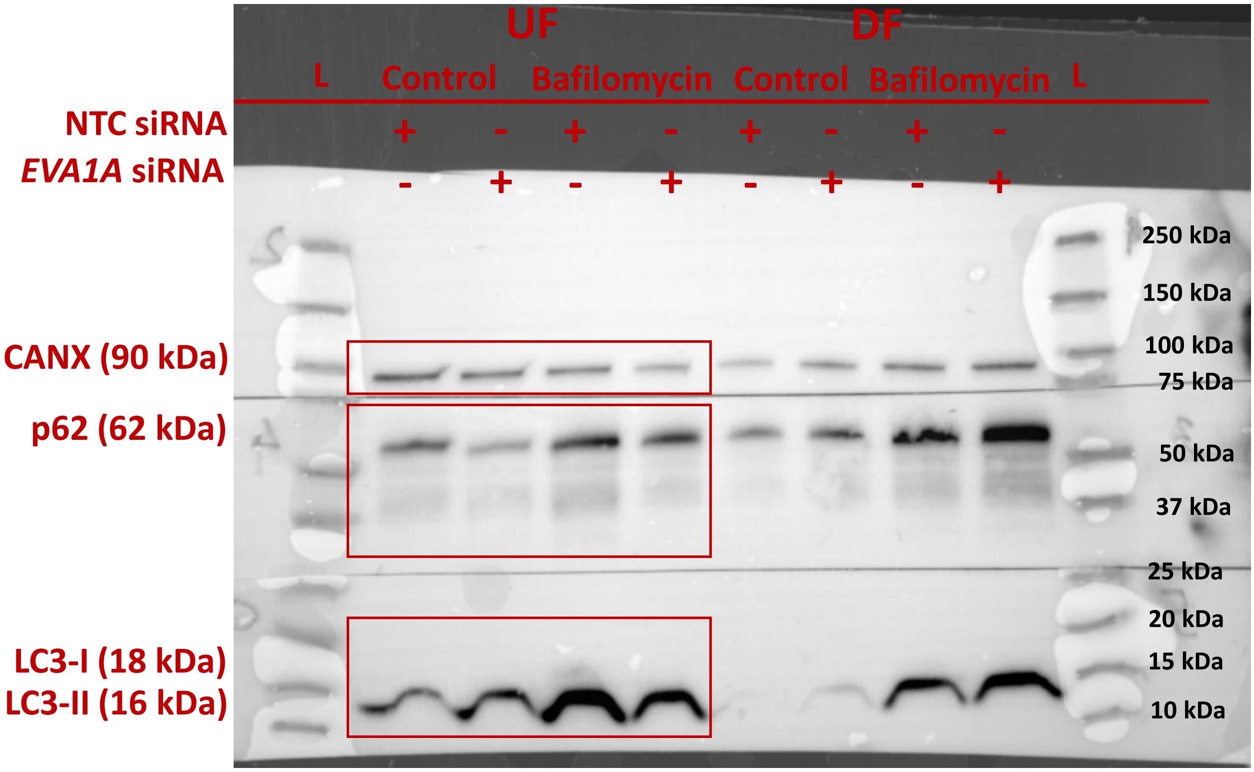

Full unedited gel for Supp. Figure 8 (2+3)

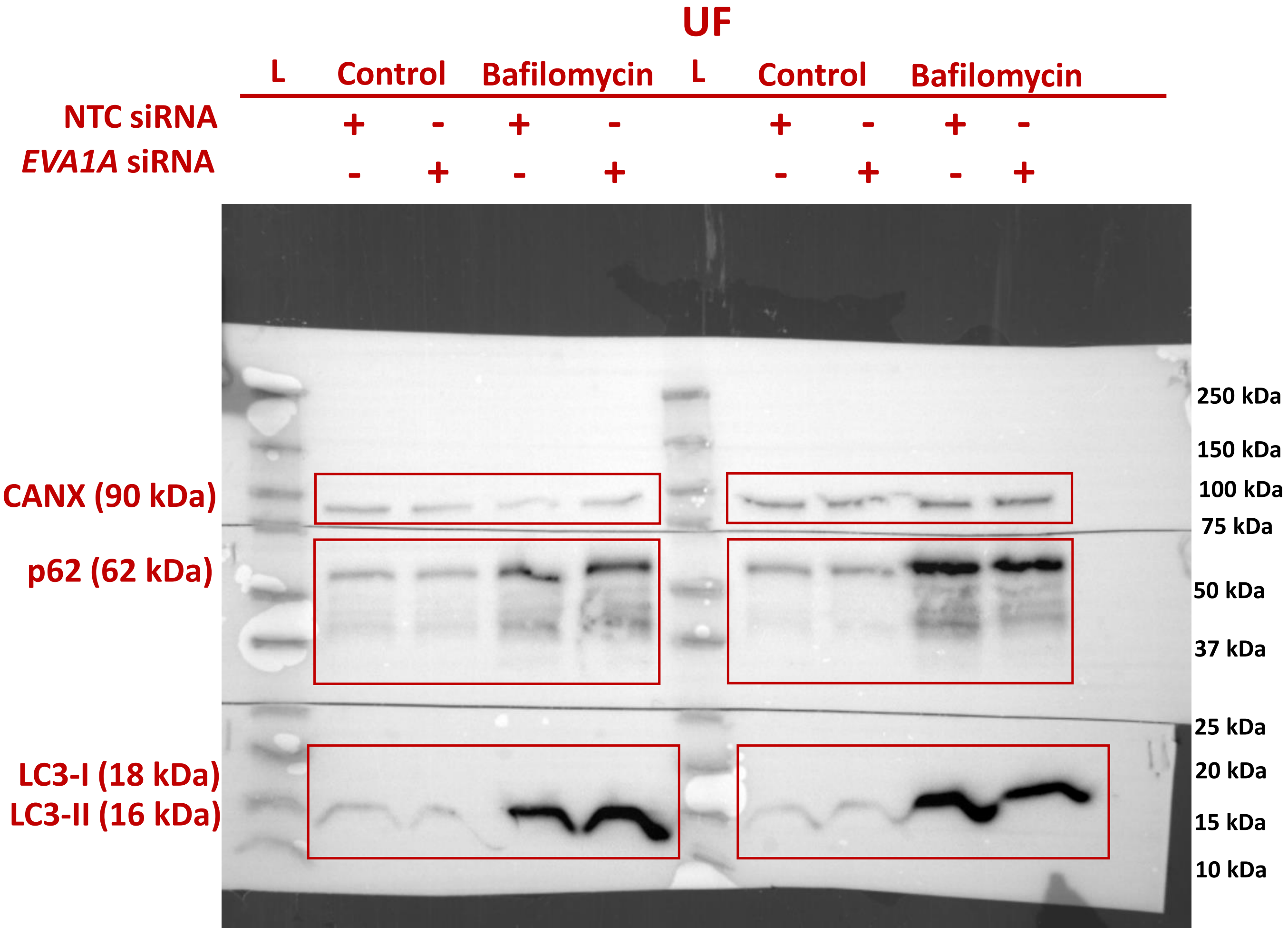

Full unedited gel for Supp. Figure 9

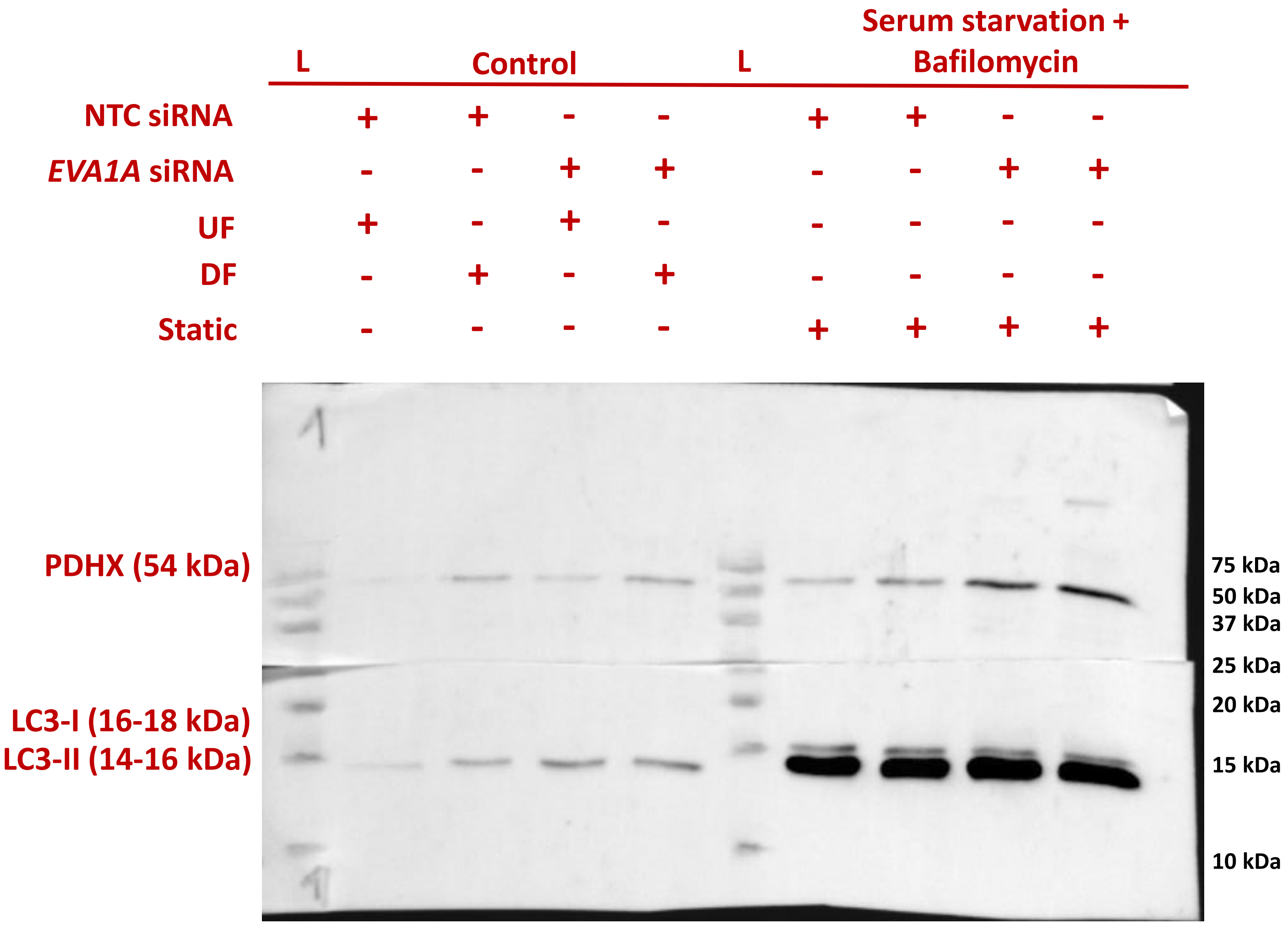

Full unedited gel for Supp. Figure 11

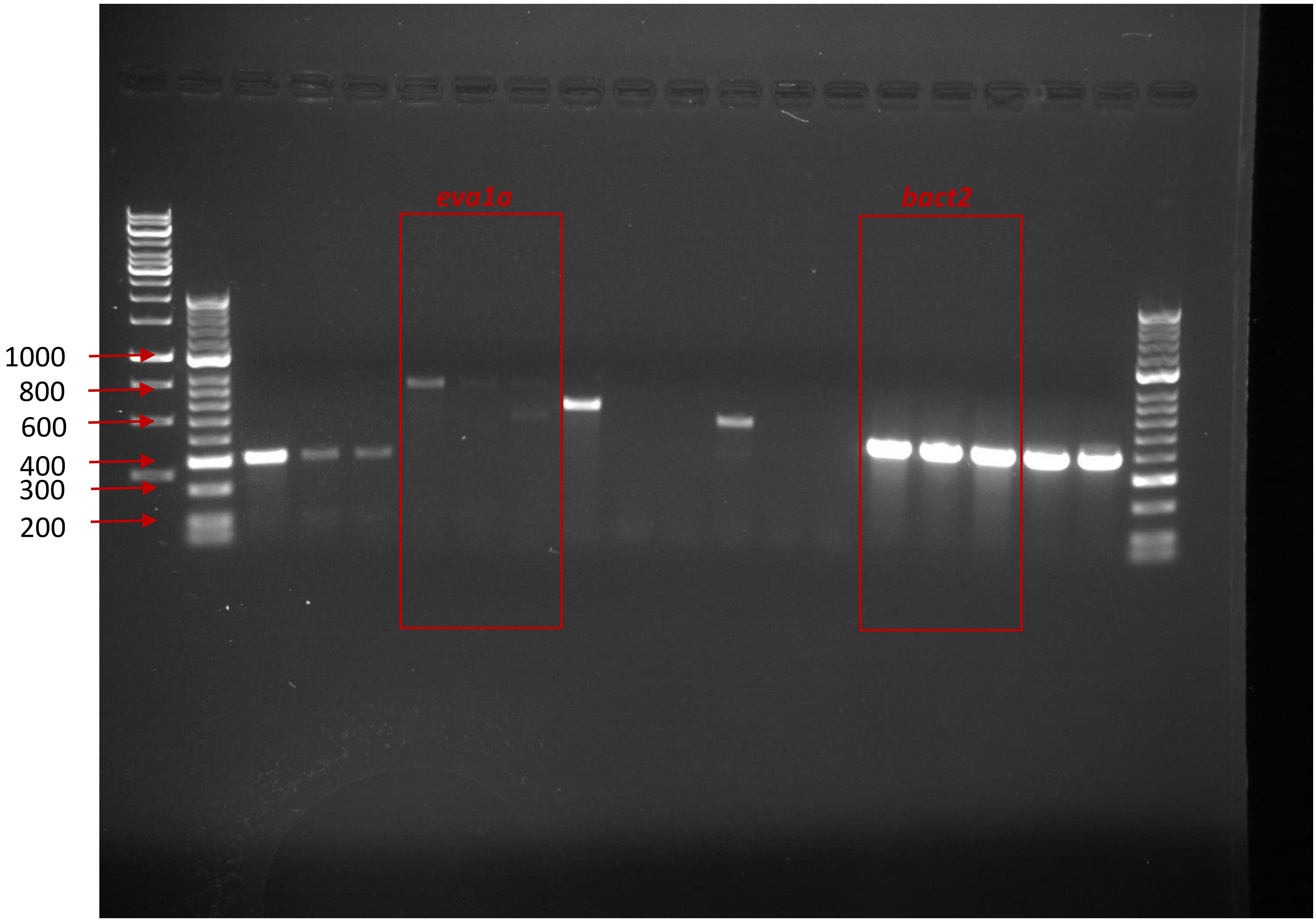

Major Resources Table

In order to allow validation and replication of experiments, all essential research materials listed in the Methods should be included in the Major Resources Table below. Authors are encouraged to use public repositories for protocols, data, code, and other materials and provide persistent identifiers and/or links to repositories when available. Authors may add or delete rows as needed.

Animals (in vivo studies)

Species	Vendor or Source	Background Strain	Sex	Persistent ID / URL
Zebrafish	University of Sheffield	AB	N/A	https://doi.org/10.1016/j.ydbio.2008.01.038
Mouse	Charles River	C57BL/6J	2 M, 2 F	https://www.criver.com/products-services/find-model/jax-c57bl6j-mice?region=3671
Pigs	Local abattoir	Unknown	N/A	N/A

Genetically Modified Animals

	Species	Vendor or Source	Background Strain	Other Information	Persistent ID / URL
**Parent - Male**	N/A	N/A	N/A	N/A	N/A
**Parent - Female**	N/A	N/A	N/A	N/A	N/A

Antibodies

Target antigen	Vendor or Source	Catalog #	Working concentration	Lot # (preferred but not required)	Persistent ID / URL
Active caspase-3	Cell Signaling	9661	2 µg/ml		
Active caspase-3	R&D Systems	AF835	5 µg/ml		
EVA1A	Proteintech	26726-1-AP	5 µg/ml		
CANX	BD Biosciences	610524	0.08 µg/ml		
CD31	Biolegend	102514	5 µg/ml		
CDH5	BD Biosciences	555661	1 µg/ml		
LC3-I, LC3-II	nanoTools	0260-100/LC3-2G6	0.5 µg/ml		
p62	BD Biosciences	610832	0.25 µg/ml		
PDHX	Santa Cruz	sc-393644	0.2 µg/ml		
VCAM1	abcam	134047	1 µg/ml		

DNA/cDNA Clones

Clone Name	Sequence	Source / Repository	Persistent ID / URL
N/A	N/A	N/A	N/A

Cultured Cells

Name	Vendor or Source	Sex (F, M, or unknown)	Persistent ID / URL
HUVEC	PromoCell	(1 F, 1 M, 4 unknown)	https://promocell.com/product/human-umbilical-vein-endothelial-cells-huvec/
HAEC	PromoCell	(1 F, 2 M)	https://promocell.com/product/human-aortic-endothelial-cells-haoec/

DOI [to be added]

Data & Code Availability

Description	Source / Repository	Persistent ID / URL
N/A	N/A	N/A

Other

Description	Source / Repository	Persistent ID / URL
N/A	N/A	N/A

DOI [to be added]

ARRIVE GUIDELINES

The ARRIVE guidelines (https://arriveguidelines.org/) are a checklist of recommendations to improve the reporting of research involving animals. Key elements of the study design should be included below to better enable readers to scrutinize the research adequately, evaluate its methodological rigor, and reproduce the methods or findings.

Study Design

**Groups**	**Sex**	**Age**	**Number (prior to experiment)**	**Number (after termination)**	**Littermates (Yes/No)**	**Other description**
Group 1 (Control)	N/A	30 hour old zebrafish embryos	25	25	No	
Group 2 (eva1a knockdown)	N/A	30 hour old zebrafish embryos	23	23	No	
Group 3 (silent heart)	N/A	30 hour old zebrafish embryos	27	27	No	
Group 4 (silent heart + eva1a knockdown)	N/A	30 hour old zebrafish embryos	24	24	No	

Sample Size: Please explain how the sample size was decided Please provide details of any a *prior* sample size calculation, if done.

Based on our previous experiments using the silent heart model and our in vitro experiments on the eva1a function in cultured human endothelial cells, we assumed a standard deviation of 20%, a power of 80% and a difference in the mean of 20%, which provides groups of 16 to reach significance.

Inclusion Criteria

N/A

Exclusion Criteria

N/A

Randomization

N/A

Blinding

The groups were labelled with nonidentifying terms before being imaged and processed by the researcher.

DOI [to be added]
